# Supplementary material for: A novel Fer/FerT targeting compound selectively evokes metabolic stress and necrotic death in malignant cells
Source: Nat Commun. 2017 Oct 16;8:940. doi: 10.1038/s41467-017-00832-w (PMC5643328; doi:10.1038/s41467-017-00832-w)
Supplement: Supplementary file 1 — Supplementary Information [file 41467_2017_832_MOESM1_ESM.pdf]

A.

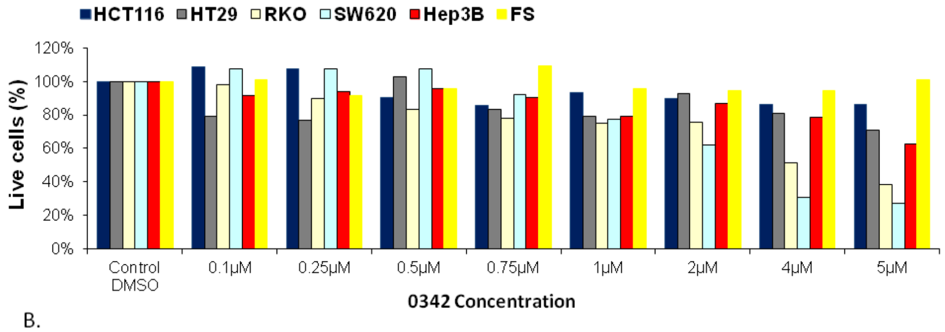
HCT116 HT29 RKO SW620 Hep3B FS
Live cells (%)
120%
100%
80%
60%
40%
20%
0%
Control DMSO
0.1μM
0.25μM
0.5μM
0.75μM
1μM
2μM
4μM
5μM
0342 Concentration

B.

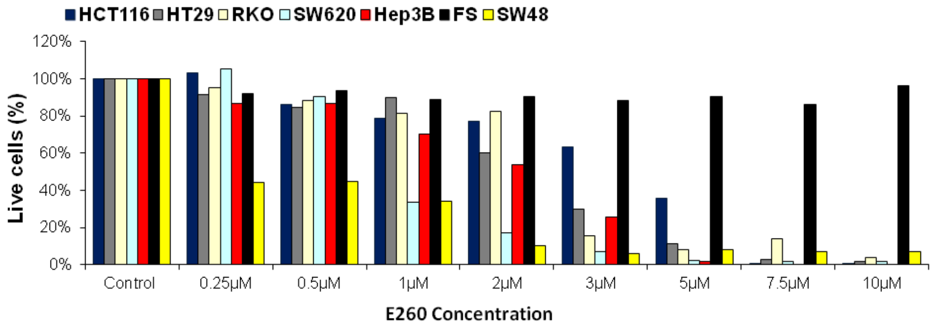
HCT116 HT29 RKO SW620 Hep3B FS SW48
Live cells (%)
120%
100%
80%
60%
40%
20%
0%
Control
0.25μM
0.5μM
1μM
2μM
3μM
5μM
7.5μM
10μM
E260 Concentration

C.

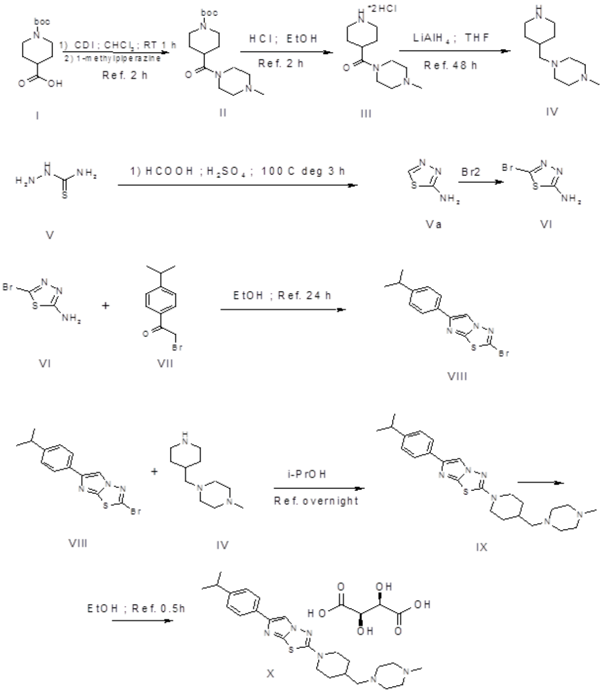
1) CDI ; CHCl3 ; RT 1 h
2) 1-methylpiperazine
Ref. 2 h
HCl ; EtOH
Ref. 2 h
LiAlH4 ; THF
Ref. 48 h
I
II
III
IV
1) HCOOH ; H2SO4 ; 100 C deg 3 h
Br2
V
Va
VI
EtOH ; Ref. 24 h
VI
VII
VIII
i-PrOH
Ref. overnight
VIII
IV
IX
EtOH ; Ref. 0.5h
X

D.

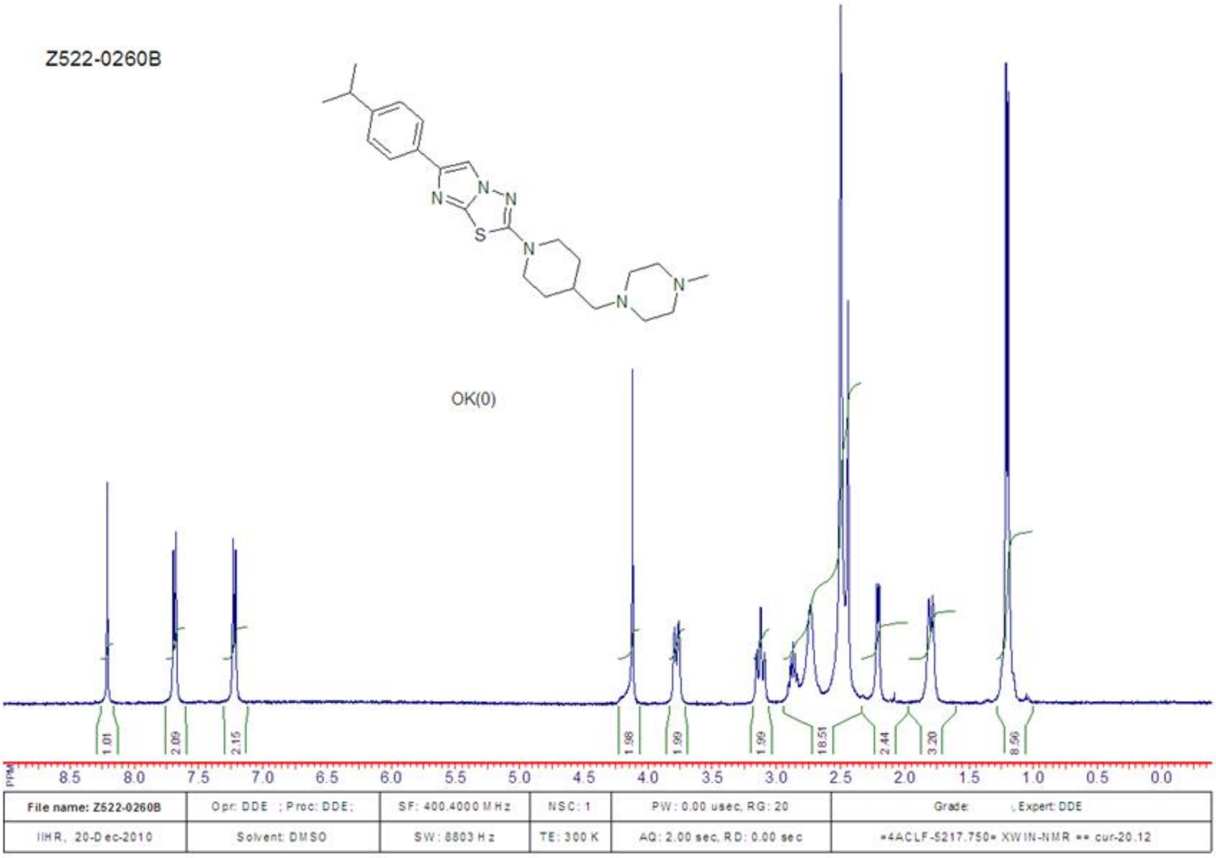

Z522-0260B
N
N
N
S
N
N
N
OK(0)
1.01
2.09
2.15
1.98
1.99
1.99
18.51
2.44
3.20
8.56
PPM
8.5
8.0
7.5
7.0
6.5
6.0
5.5
5.0
4.5
4.0
3.5
3.0
2.5
2.0
1.5
1.0
0.5
0.0
File name: Z522-0260B
Opr: DDE ; Proc: DDE;
SF: 400.4000 MHz
NSC: 1
PW: 0.00 usec, RG: 20
Grade: , Expert: DDE
IIHR, 20-Dec-2010
Solvent: DMSO
SW: 8803 Hz
TE: 300 K
AQ: 2.00 sec, RD: 0.00 sec
=4ACLF-5217.750= XWIN-NMR == cur-20.12

E.

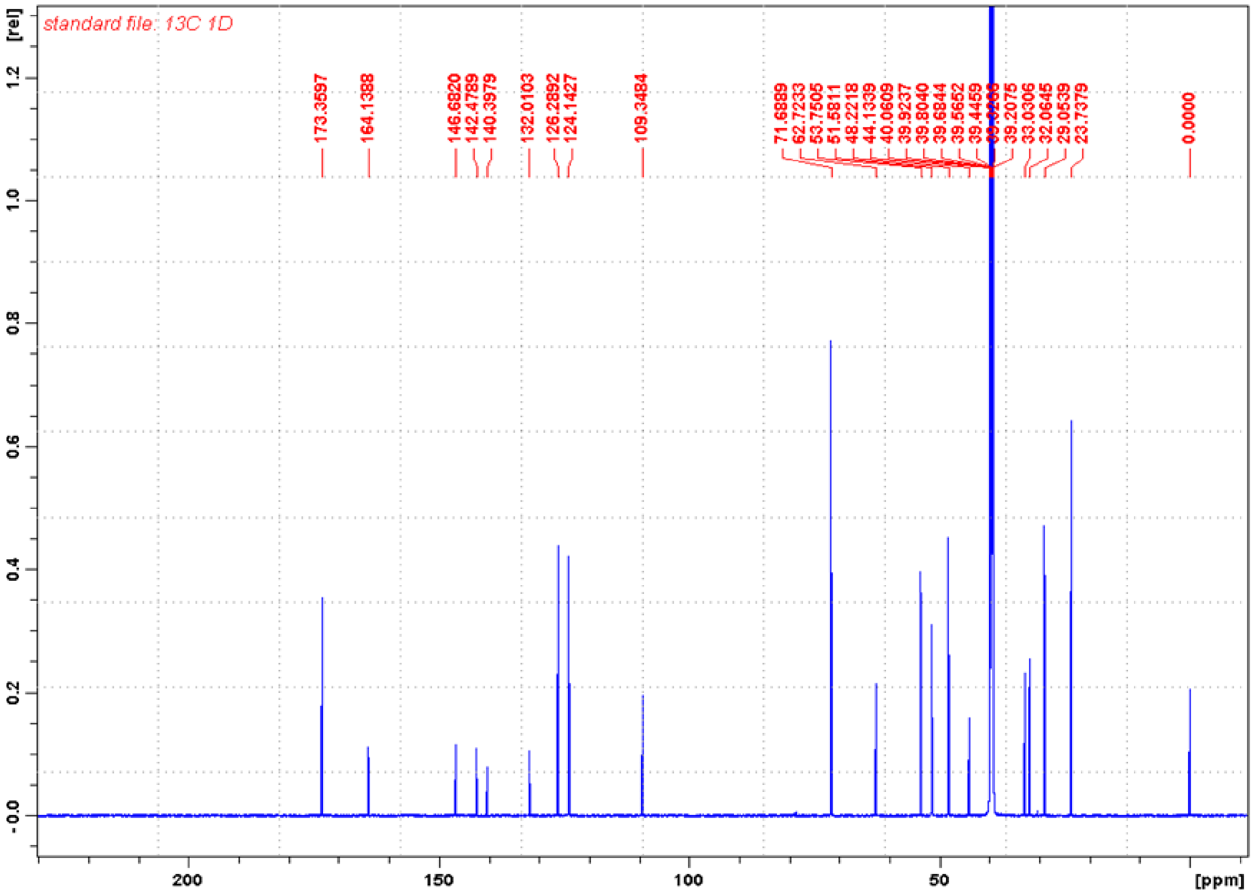
standard file: 13C 1D
[rel]
1.2
1.0
0.8
0.6
0.4
0.2
-0.0
173.3597
164.1388
146.6820
142.4789
140.3979
132.0103
126.2892
124.1427
109.3484
71.6889
62.7233
53.7505
51.5811
48.2218
44.1339
40.0609
39.9237
39.8040
39.6844
39.5652
39.4459
39.2075
33.0306
32.0645
29.0539
23.7379
0.0000
200
150
100
50
[ppm]

F.

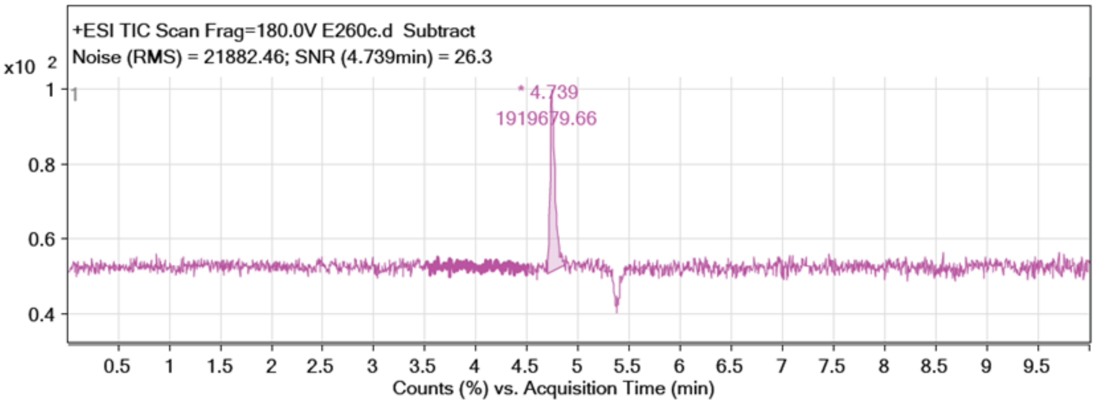

+ESI TIC Scan Frag=180.0V E260c.d Subtract
Noise (RMS) = 21882.46; SNR (4.739min) = 26.3
x10 2
* 4.739
1919679.66
1
0.8
0.6
0.4
0.5 1 1.5 2 2.5 3 3.5 4 4.5 5 5.5 6 6.5 7 7.5 8 8.5 9 9.5
Counts (%) vs. Acquisition Time (min)

G.

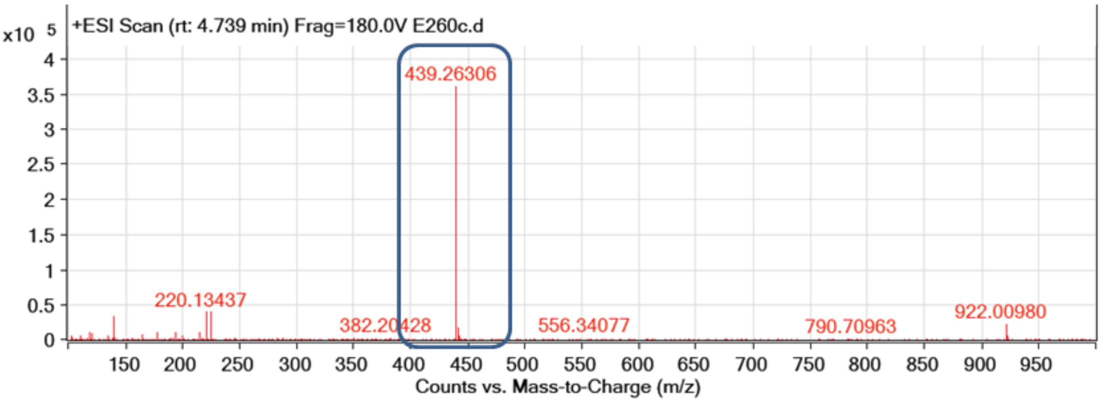

+ESI Scan (rt: 4.739 min) Frag=180.0V E260c.d
x10 5
439.26306
220.13437
382.20428
556.34077
790.70963
922.00980
4
3.5
3
2.5
2
1.5
1
0.5
0
150 200 250 300 350 400 450 500 550 600 650 700 750 800 850 900 950
Counts vs. Mass-to-Charge (m/z)

H.

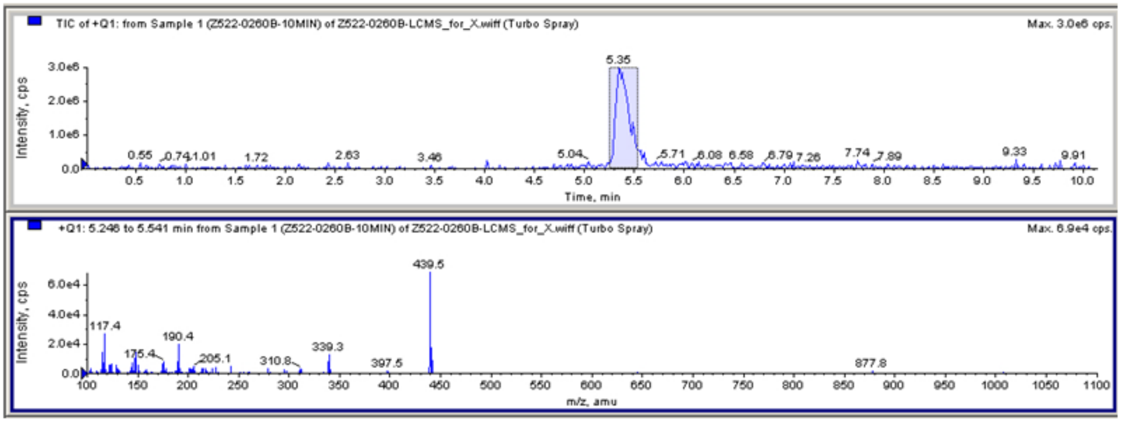

TIC of +Q1: from Sample 1 (Z522-0260B-10MIN) of Z522-0260B-LCMS_for_X.wiff (Turbo Spray)
Max. 3.0e6 cps.
Intensity, cps
5.35
Time, min
+Q1: 5.246 to 5.541 min from Sample 1 (Z522-0260B-10MIN) of Z522-0260B-LCMS_for_X.wiff (Turbo Spray)
Max. 6.9e4 cps.
439.5
117.4
190.4
339.3
397.5
877.8
m/z, amu

I.

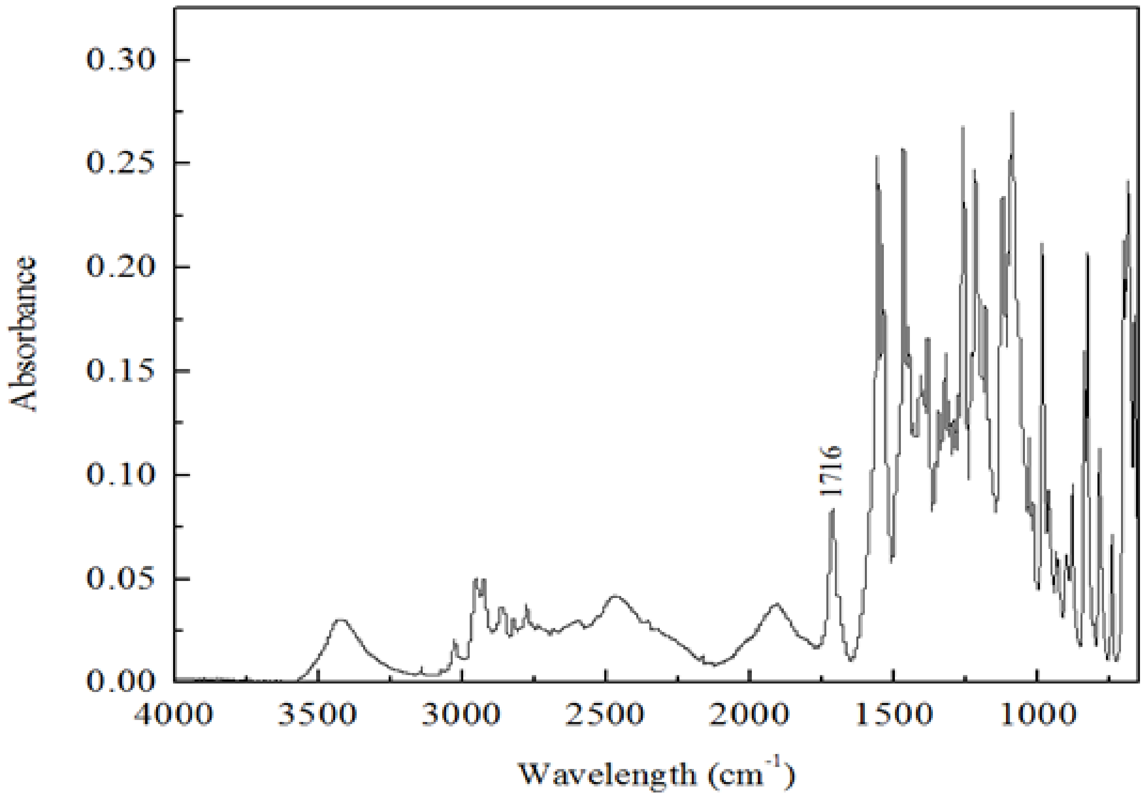

0.30
0.25
0.20
0.15
0.10
0.05
0.00
Absorbance
1716
4000
3500
3000
2500
2000
1500
1000
Wavelength (cm-1)

Supplementary figure 1. Cell-viability based XTT HTS system for optimization of the Fer inhibitor E260 and its synthesis process. XTT based robotic HTS analysis depicting the viability of cells incubated for 96h with ascending concentrations of (**A**) 0342, or (**B**) E260, dissolved in DMSO (Not formulated in Cremophor EL ®); n=2. (**C**) The complete synthesis scheme of the Fer inhibitor E260. (**D**) ^{1}H-NMR and (**E**) ^{13}C-NMR spectras of the synthesized E260 compound. For **D** Integral of peaks reflects number of protons. Also this spectrum contains signal of tartrate. Confirming a purity of >95%. (**F**) UPLC analysis of the compound E260 with a retention time of 4.73 minutes and purity of >99%. (**G**) HRMS analysis of the compound E260 confirms a molecular weight of 439.263 Dalton as calculated (Boxed with blue Square). Second peak at 220.136 depicts E260 which was charged with two protons. (**H**) LCMS of the compound E260 with a retention time of 5.35 minutes, molecular weight of 439.5 Dalton and purity of >95%.(**I**) FTIR spectrum of E260-tartrate. The peak at 1716 cm^{-1} corresponds to the carbonyl of the tartrate salt.

A.

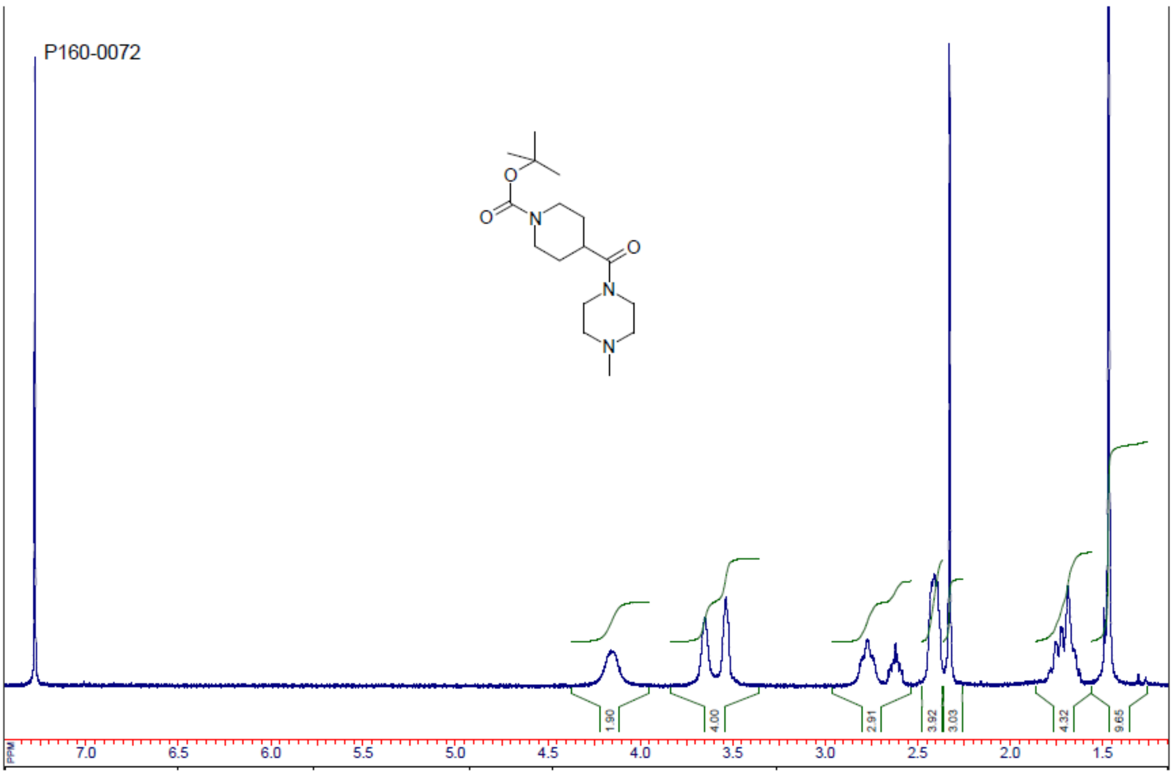

P160-0072
1.90
4.00
2.91
3.92
3.03
4.32
9.65
PPM
7.0
6.5
6.0
5.5
5.0
4.5
4.0
3.5
3.0
2.5
2.0
1.5

B.

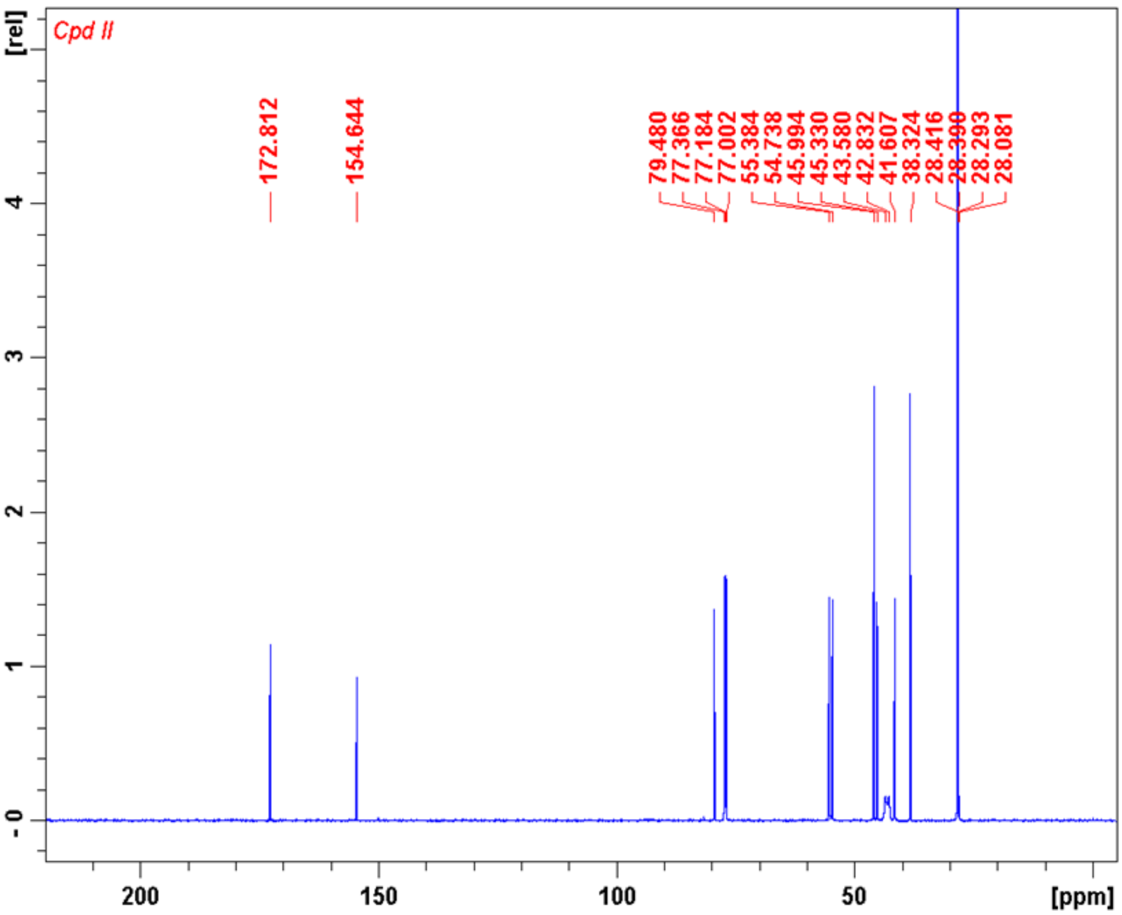
Cpd II
[rel]
172.812
154.644
79.480
77.366
77.184
77.002
55.384
54.738
45.994
45.330
43.580
42.832
41.607
38.324
28.416
28.390
28.293
28.081
0
1
2
3
4
200
150
100
50
[ppm]

C.

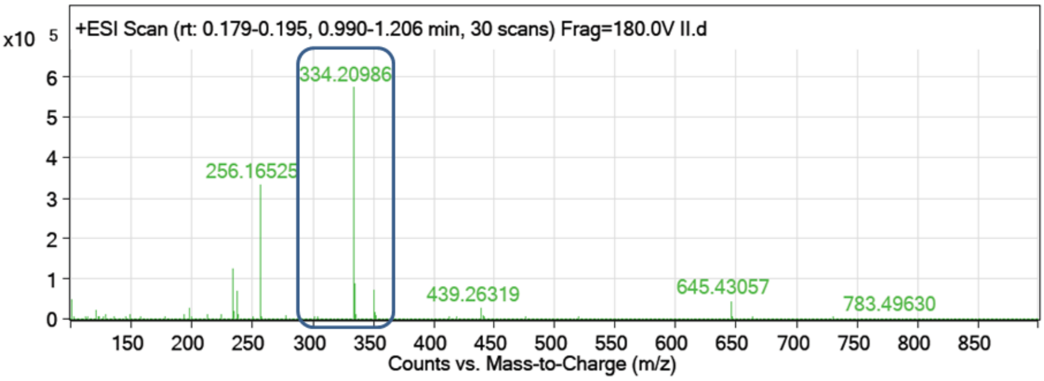

x10 5
+ESI Scan (rt: 0.179-0.195, 0.990-1.206 min, 30 scans) Frag=180.0V II.d
334.20986
256.16525
439.26319
645.43057
783.49630
0
1
2
3
4
5
6
150
200
250
300
350
400
450
500
550
600
650
700
750
800
850
Counts vs. Mass-to-Charge (m/z)

D.

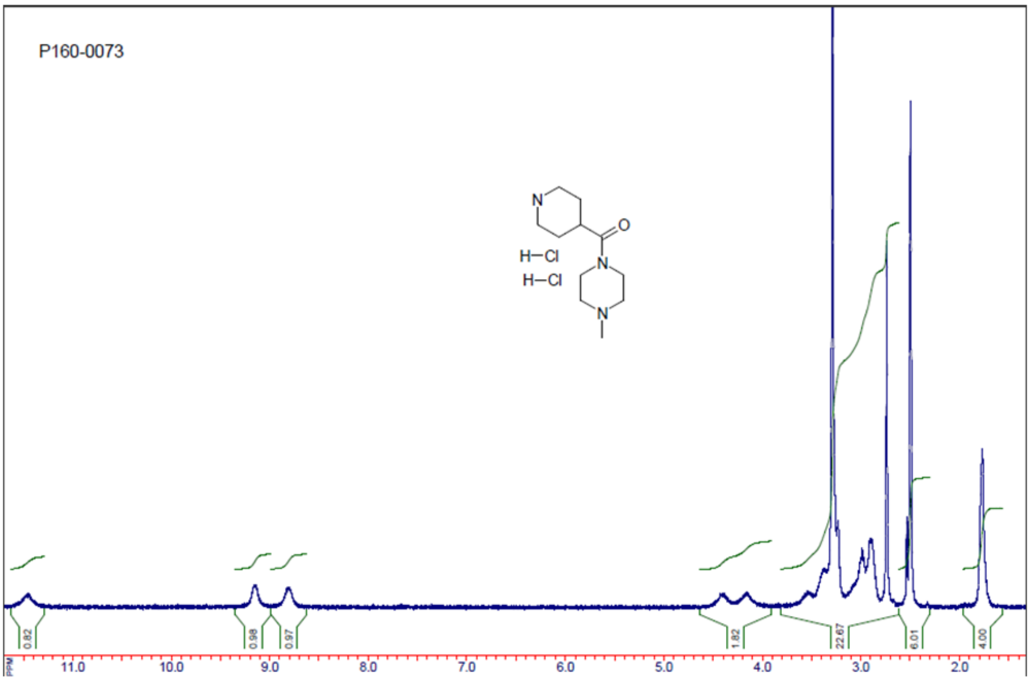
P160-0073
H—Cl
H—Cl
0.82
0.98
0.97
1.82
22.67
6.01
4.00
PPM
11.0
10.0
9.0
8.0
7.0
6.0
5.0
4.0
3.0
2.0

E.

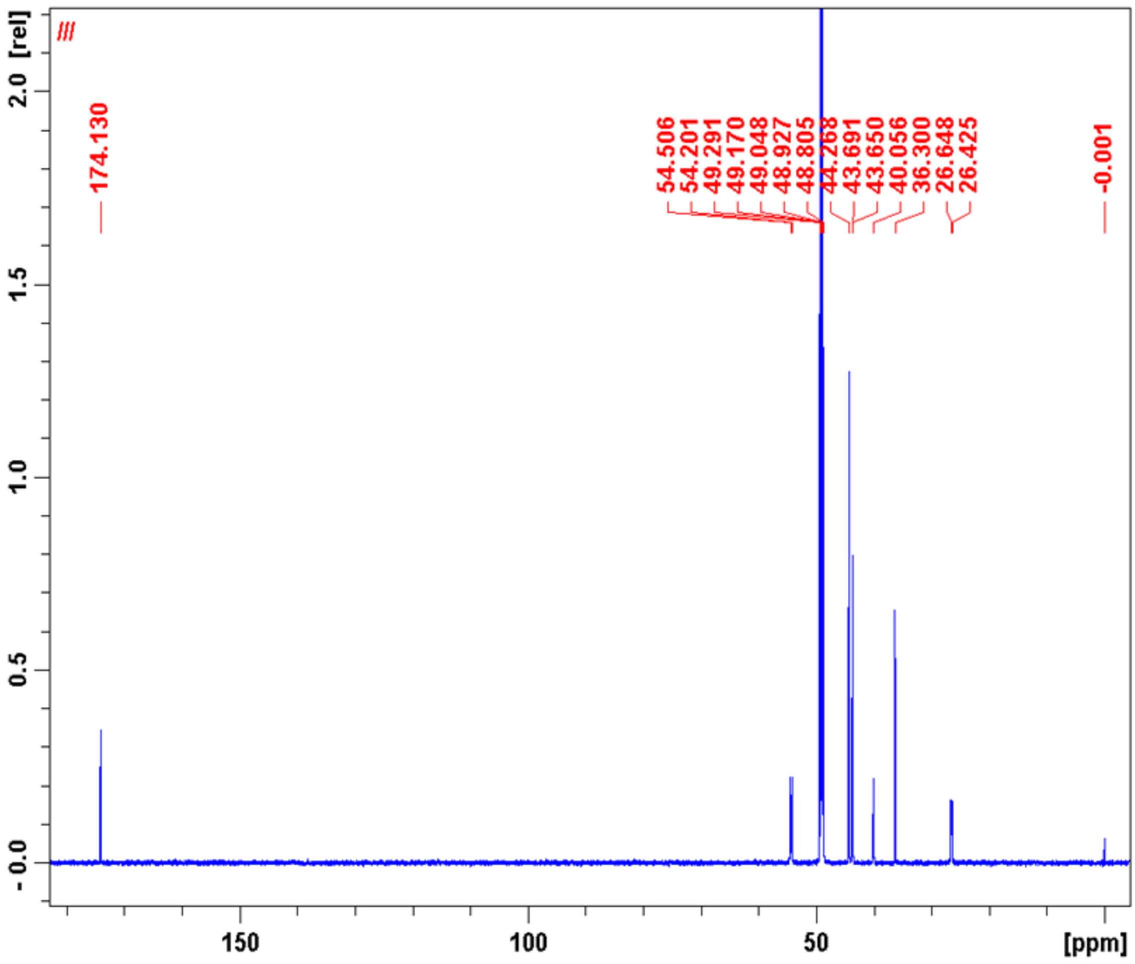
[rel]
2.0
1.5
1.0
0.5
- 0.0
174.130
54.506
54.201
49.291
49.170
49.048
48.927
48.805
44.268
43.691
43.650
40.056
36.300
26.648
26.425
-0.001
150
100
50
[ppm]

F.

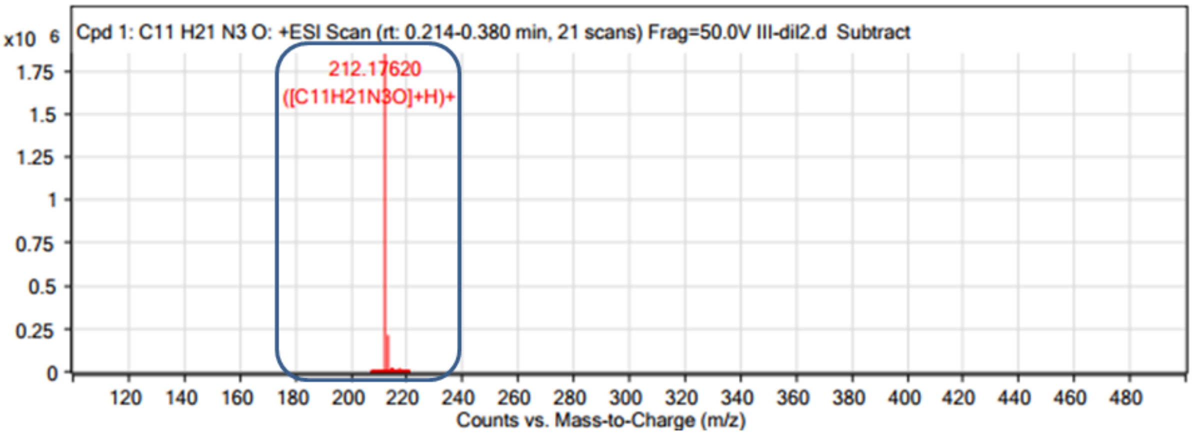
Cpd 1: C11 H21 N3 O: +ESI Scan (rt: 0.214-0.380 min, 21 scans) Frag=50.0V III-dil2.d Subtract
x10 6
212.17620
([C11H21N3O]+H)+
1.75
1.5
1.25
1
0.75
0.5
0.25
0
120 140 160 180 200 220 240 260 280 300 320 340 360 380 400 420 440 460 480
Counts vs. Mass-to-Charge (m/z)

G.

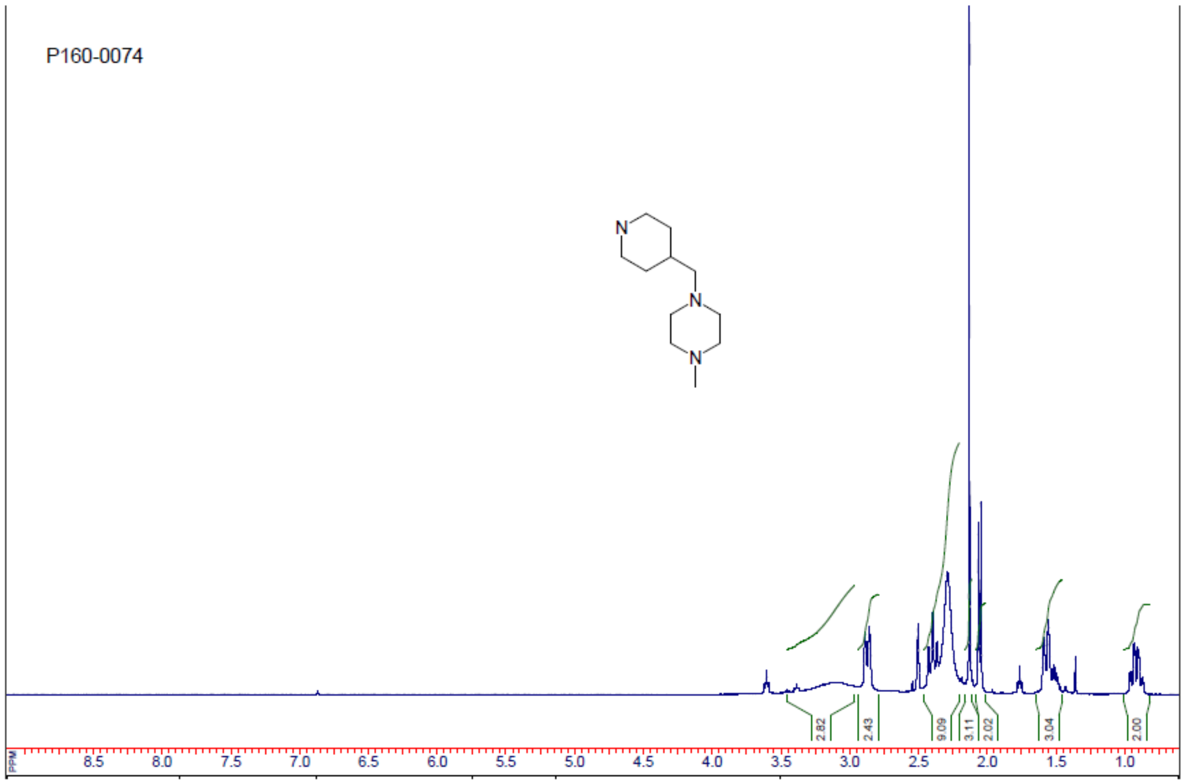

P160-0074
2.82
2.43
9.09
3.11
2.02
3.04
2.00
PPM
8.5
8.0
7.5
7.0
6.5
6.0
5.5
5.0
4.5
4.0
3.5
3.0
2.5
2.0
1.5
1.0

H.

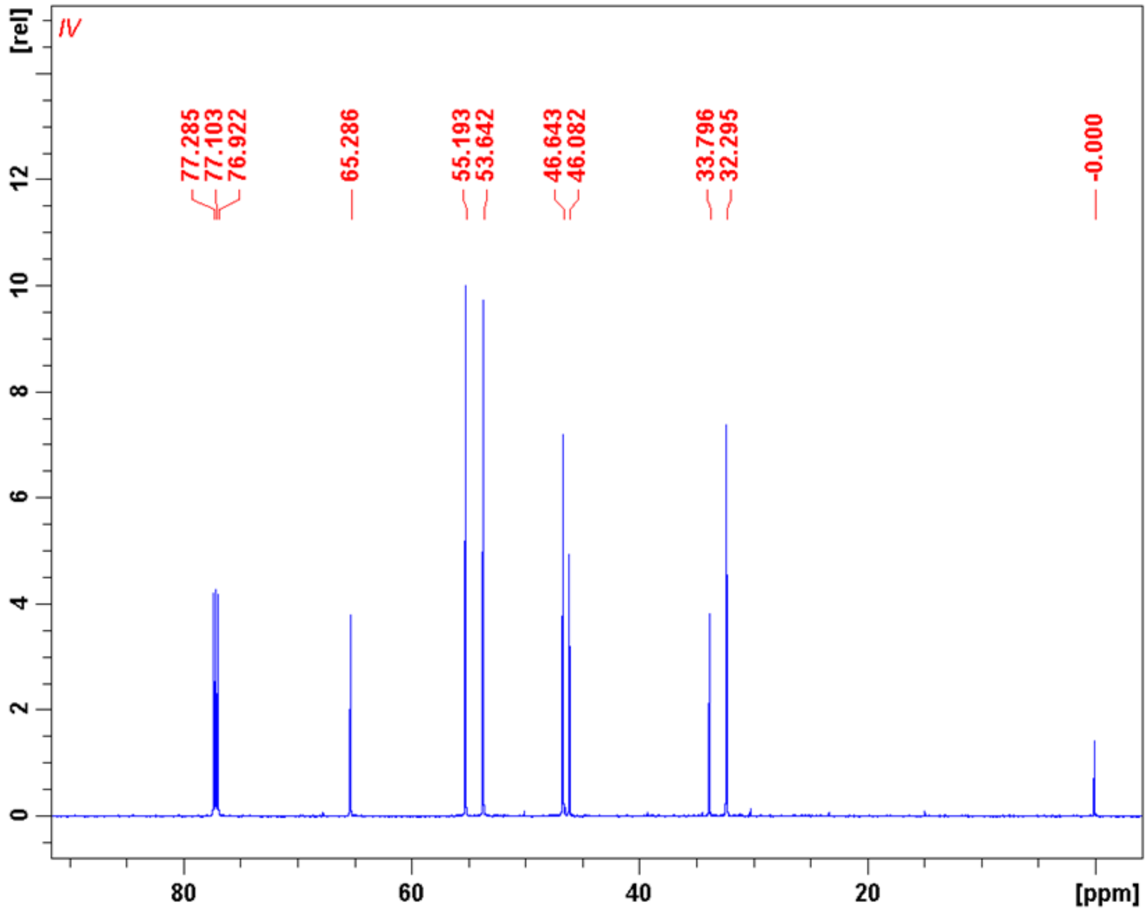

IV
[rel]
77.285
77.103
76.922
65.286
55.193
53.642
46.643
46.082
33.796
32.295
-0.000
0
2
4
6
8
10
12
80
60
40
20
[ppm]

I.

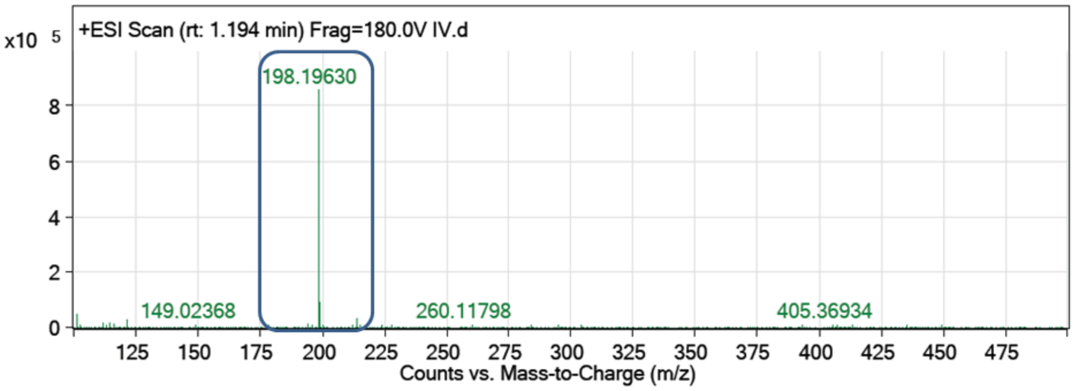

+ESI Scan (rt: 1.194 min) Frag=180.0V IV.d
x10 5
198.19630
149.02368
260.11798
405.36934
Counts vs. Mass-to-Charge (m/z)

J.

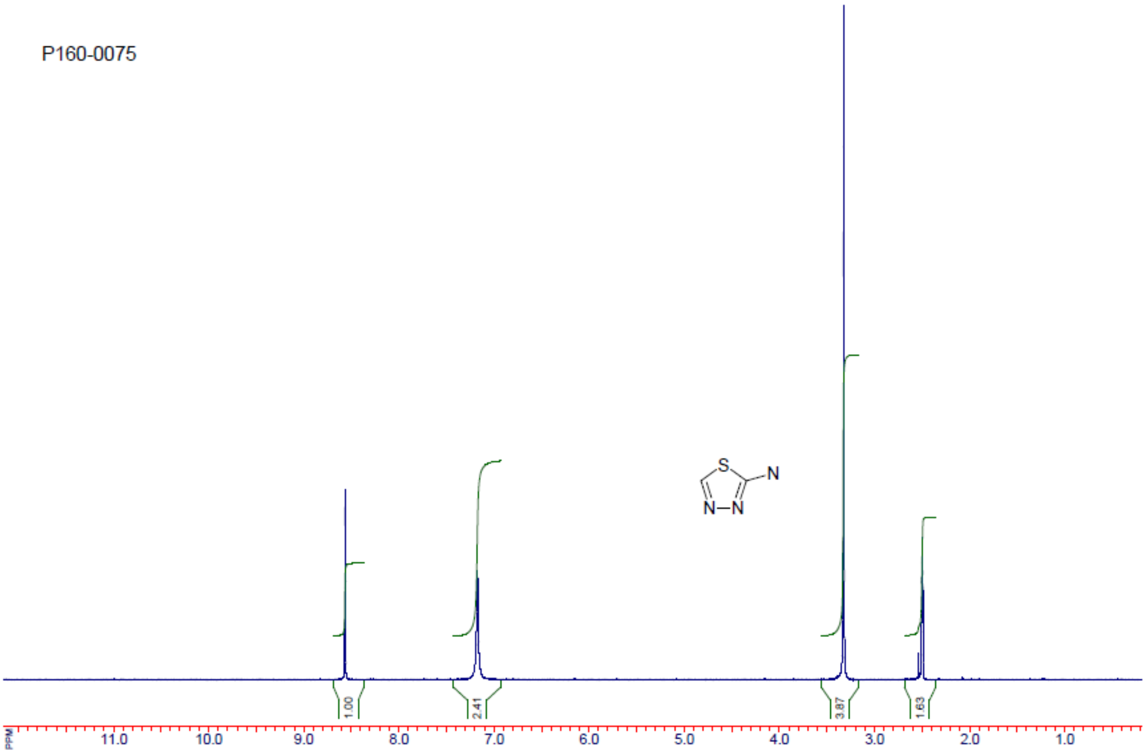
P160-0075
S
N
N–N
1.00
2.41
3.87
1.63
PPM
11.0
10.0
9.0
8.0
7.0
6.0
5.0
4.0
3.0
2.0
1.0

K.

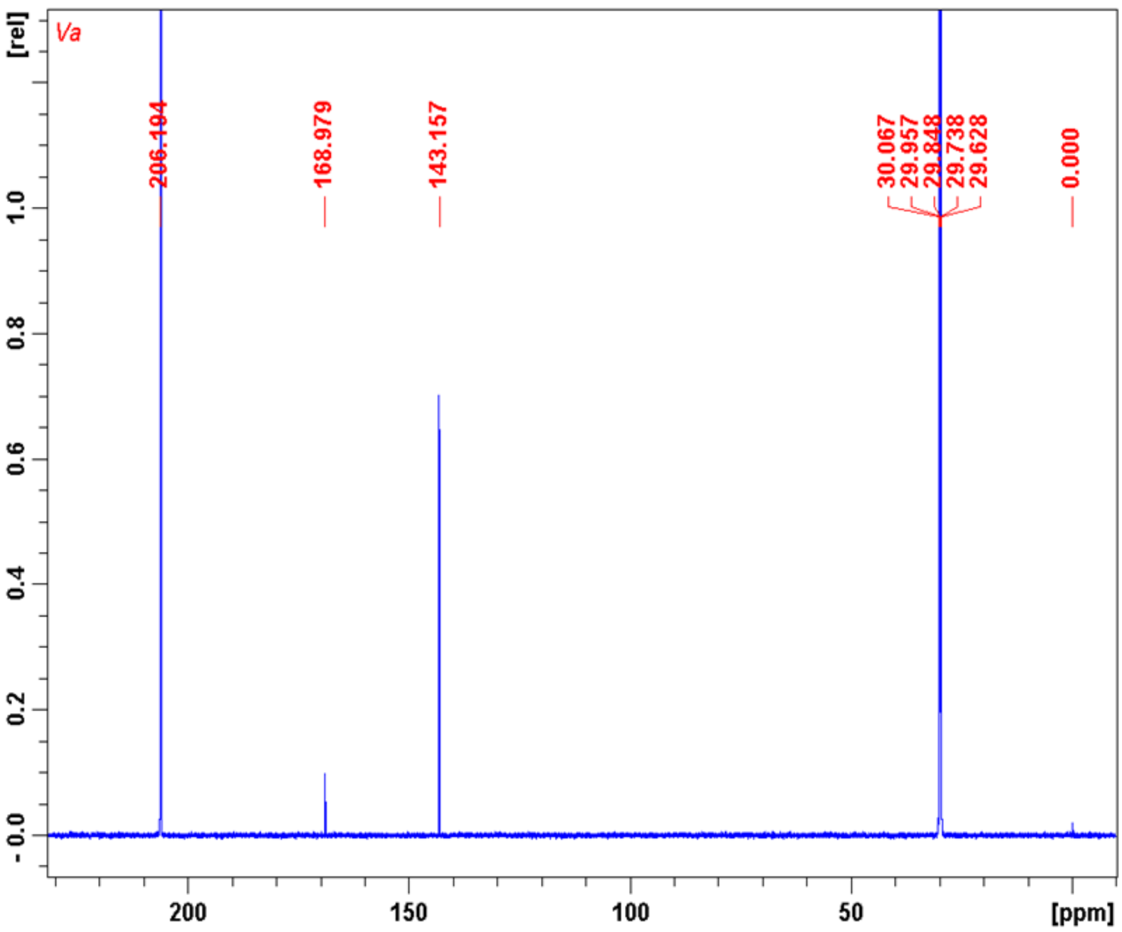

[rel]
Va
206.194
168.979
143.157
30.067
29.957
29.848
29.738
29.628
0.000
1.0
0.8
0.6
0.4
0.2
- 0.0
200
150
100
50
[ppm]

L.

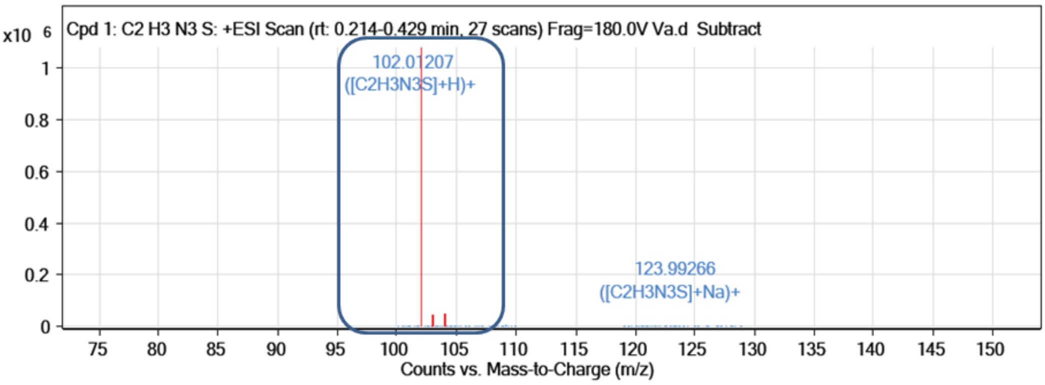

Cpd 1: C2 H3 N3 S: +ESI Scan (rt: 0.214-0.429 min, 27 scans) Frag=180.0V Va.d Subtract
x10 6
1
0.8
0.6
0.4
0.2
0
102.01207
([C2H3N3S]+H)+
123.99266
([C2H3N3S]+Na)+
75
80
85
90
95
100
105
110
115
120
125
130
135
140
145
150
Counts vs. Mass-to-Charge (m/z)

M.

P160-0076

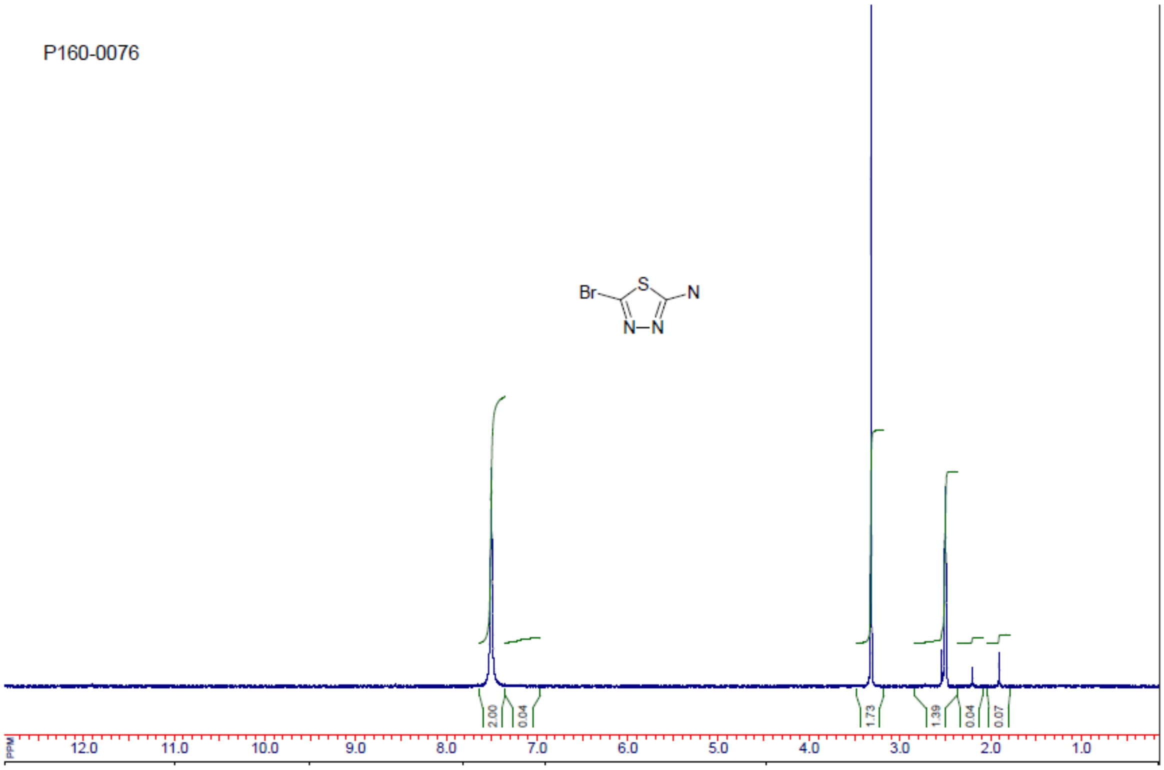
Br
S
N
N–N
2.00
0.04
1.73
1.39
0.04
0.07
PPM
12.0
11.0
10.0
9.0
8.0
7.0
6.0
5.0
4.0
3.0
2.0
1.0

N.

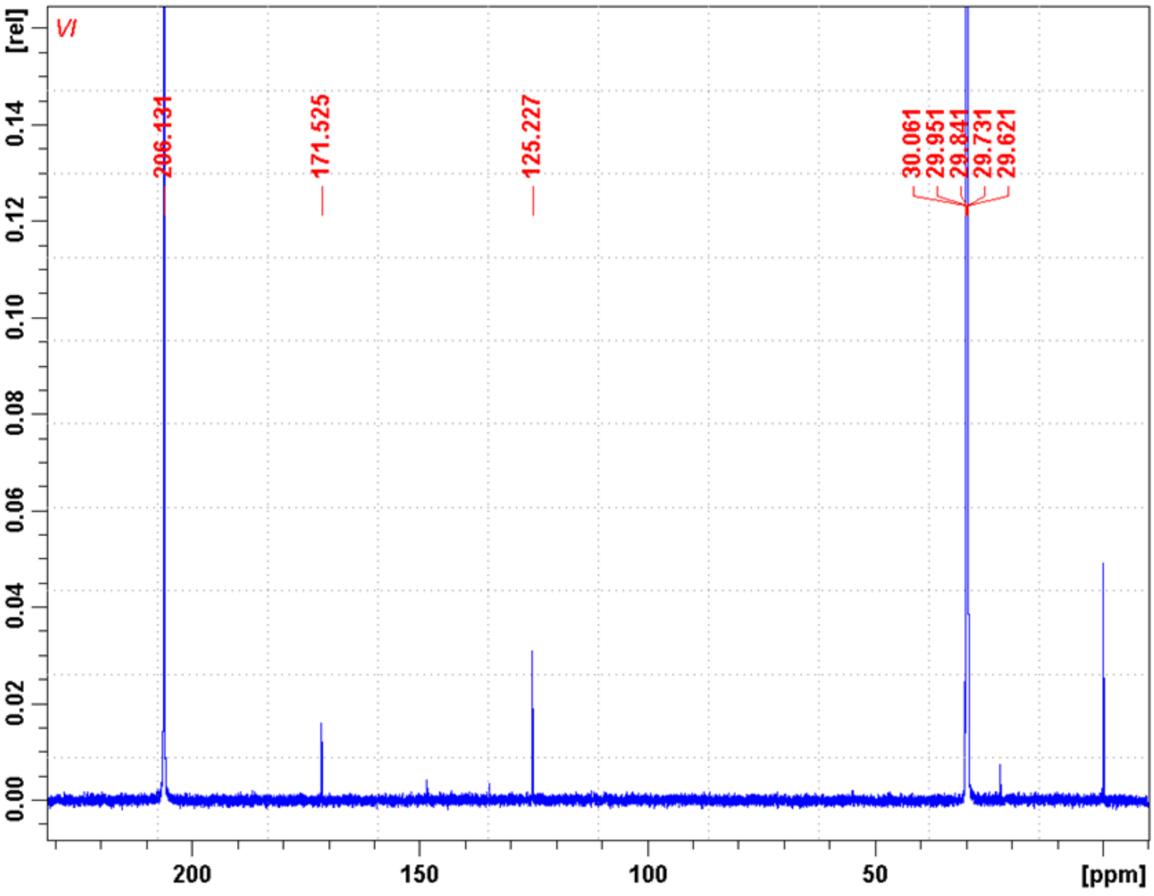
VI
206.131
171.525
125.227
30.061
29.951
29.841
29.731
29.621
[rel]
0.14
0.12
0.10
0.08
0.06
0.04
0.02
0.00
200
150
100
50
[ppm]

O.

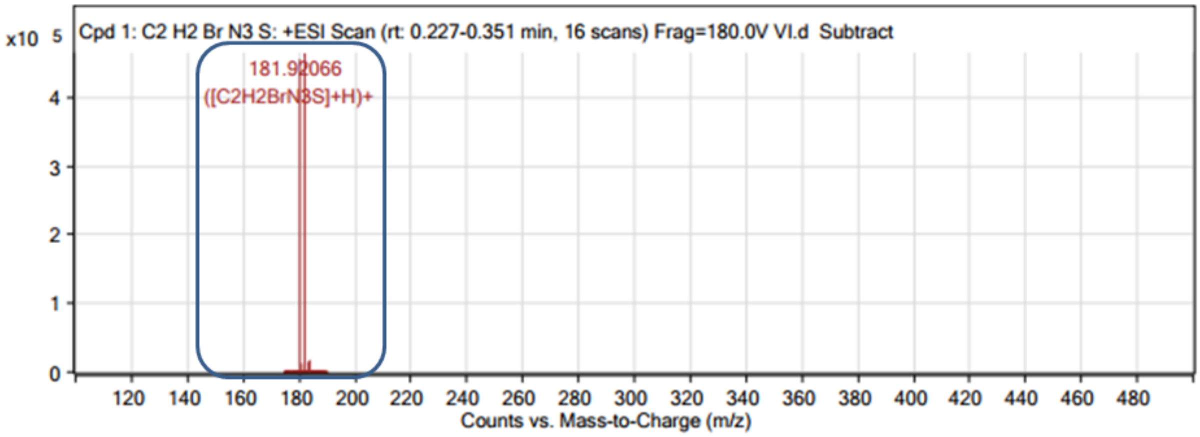
Cpd 1: C2 H2 Br N3 S: +ESI Scan (rt: 0.227-0.351 min, 16 scans) Frag=180.0V VI.d Subtract
x10 5
181.92066
([C2H2BrN3S]+H)+
Counts vs. Mass-to-Charge (m/z)

P.

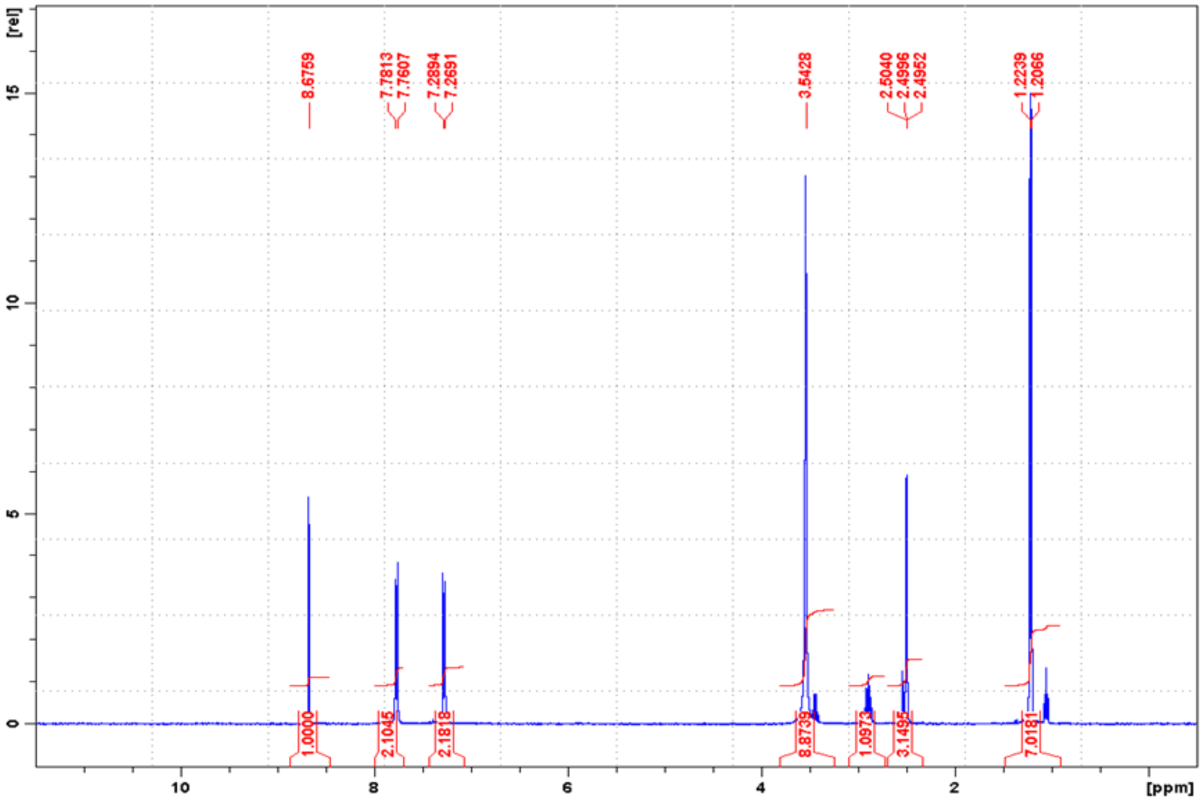

[rel]
15
10
5
0
8.6759
7.7813
7.7607
7.2894
7.2691
3.5428
2.5040
2.4996
2.4952
1.2239
1.2066
1.0000
2.1045
2.1818
8.8739
1.0973
3.1495
7.0181
10
8
6
4
2
[ppm]

Q.

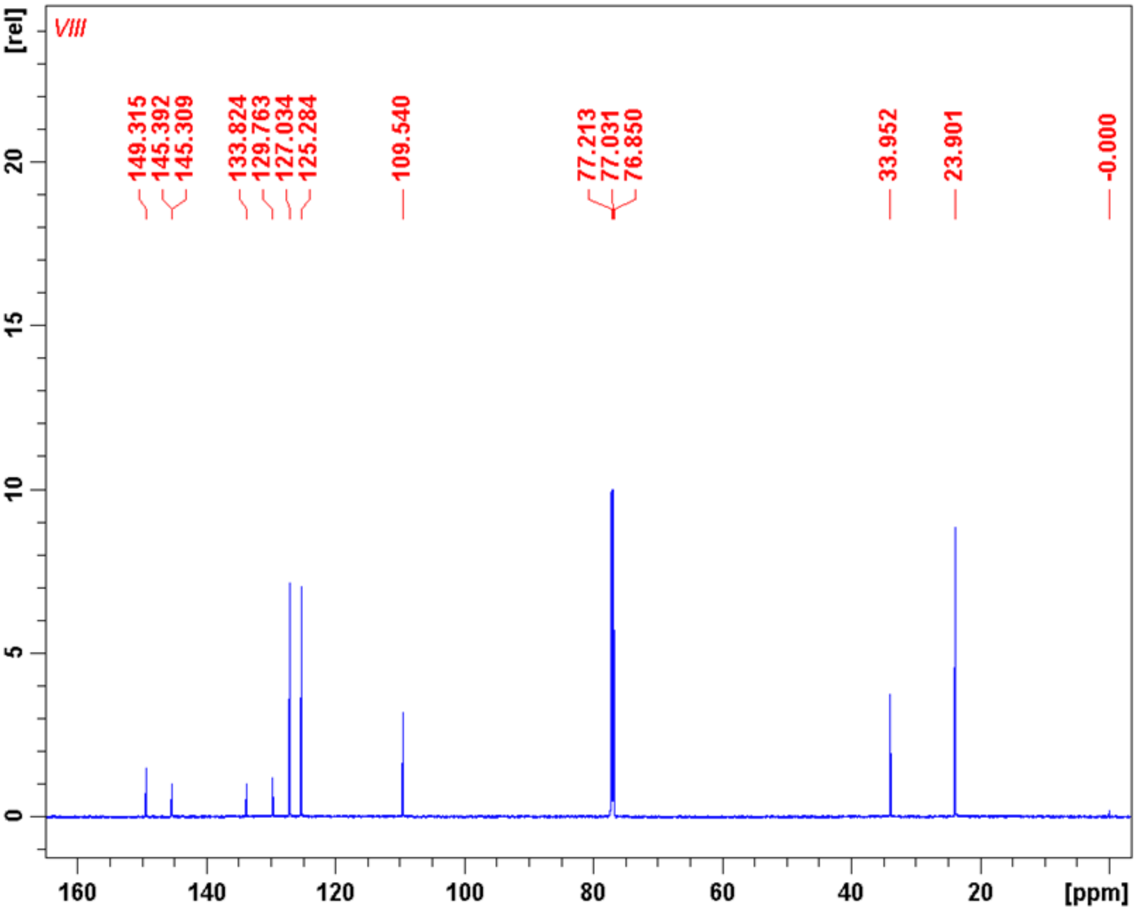

VIII
149.315
145.392
145.309
133.824
129.763
127.034
125.284
109.540
77.213
77.031
76.850
33.952
23.901
-0.000
[rel]
20
15
10
5
0
160
140
120
100
80
60
40
20
[ppm]

R.

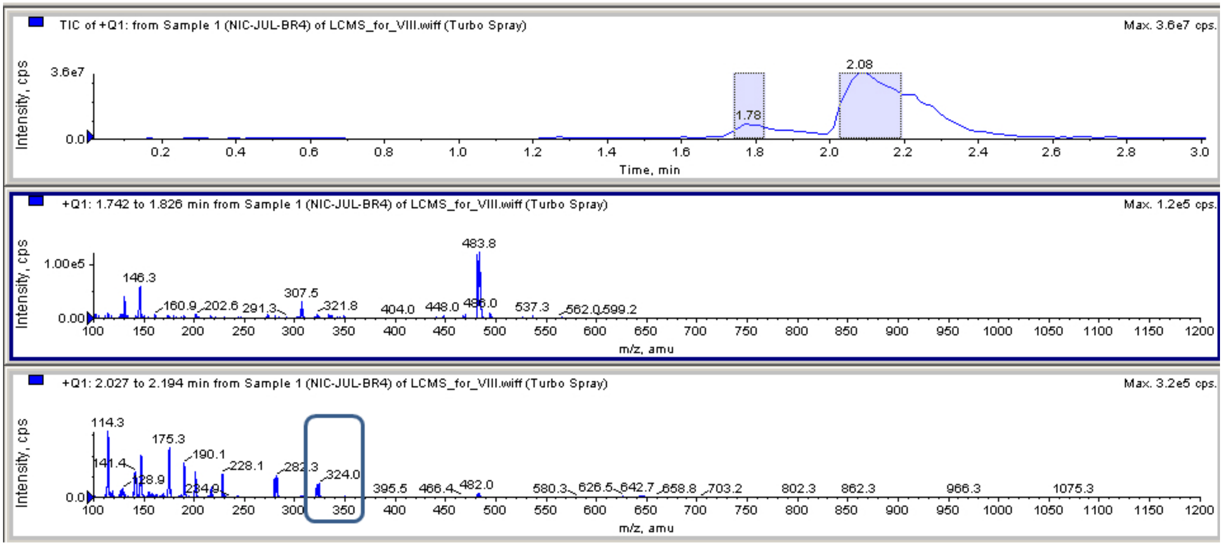
TIC of +Q1: from Sample 1 (NIC-JUL-BR4) of LCMS_for_VIII.wiff (Turbo Spray)
Max. 3.6e7 cps.
Intensity, cps
Time, min
2.08
1.78
+Q1: 1.742 to 1.826 min from Sample 1 (NIC-JUL-BR4) of LCMS_for_VIII.wiff (Turbo Spray)
Max. 1.2e5 cps.
483.8
146.3
307.5
m/z, amu
+Q1: 2.027 to 2.194 min from Sample 1 (NIC-JUL-BR4) of LCMS_for_VIII.wiff (Turbo Spray)
Max. 3.2e5 cps.
114.3
175.3
190.1
228.1
282.3
324.0
m/z, amu

S.

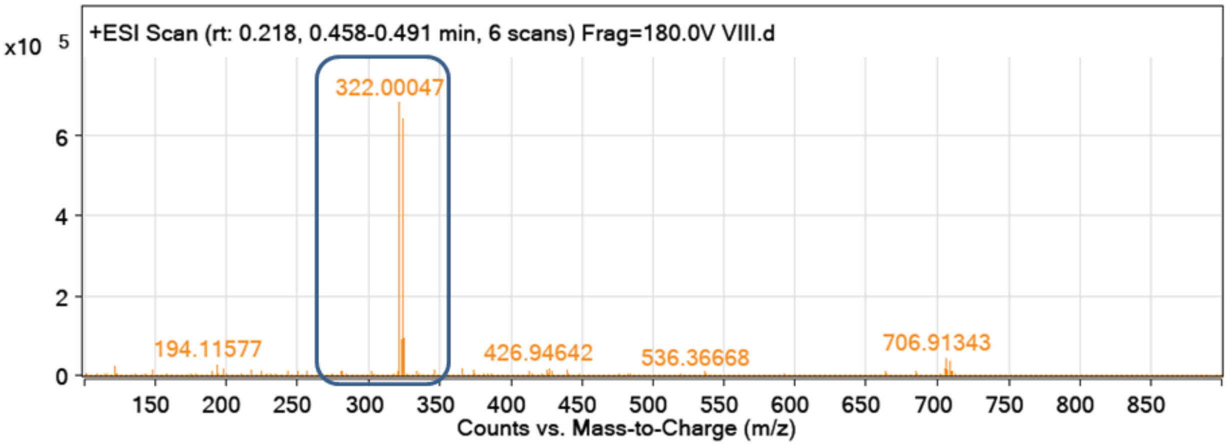
+ESI Scan (rt: 0.218, 0.458-0.491 min, 6 scans) Frag=180.0V VIII.d
x10 5
322.00047
194.11577
426.94642
536.36668
706.91343
Counts vs. Mass-to-Charge (m/z)

T.

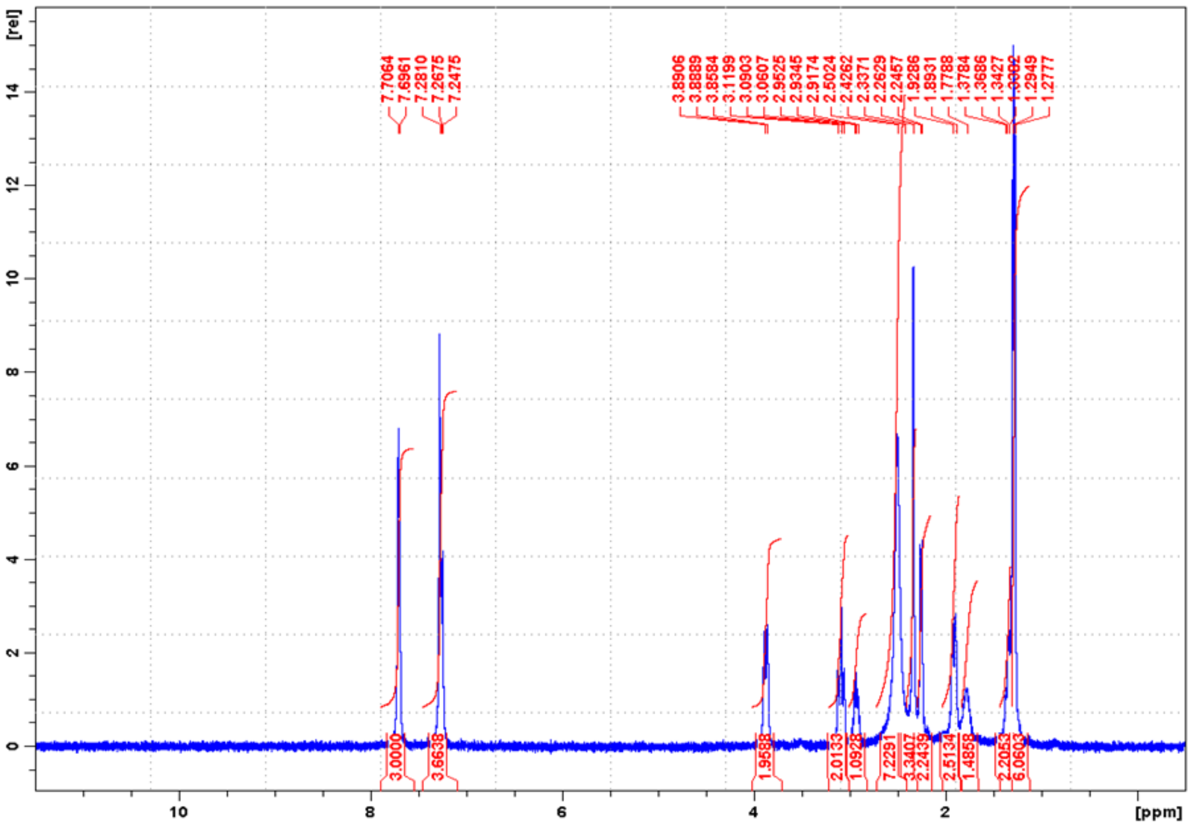

[rel]
14
12
10
8
6
4
2
0
7.7064
7.6961
7.2810
7.2675
7.2475
3.8906
3.8889
3.8584
3.1199
3.0903
3.0607
2.9525
2.9345
2.9174
2.5024
2.4262
2.3371
2.2629
2.2457
1.9286
1.8931
1.7788
1.3784
1.3686
1.3427
1.2949
1.2777
3.0000
3.6638
1.9588
2.0133
1.0928
7.2291
3.3407
2.2439
2.5134
1.4858
2.2053
6.0603
10
8
6
4
2
[ppm]

U.

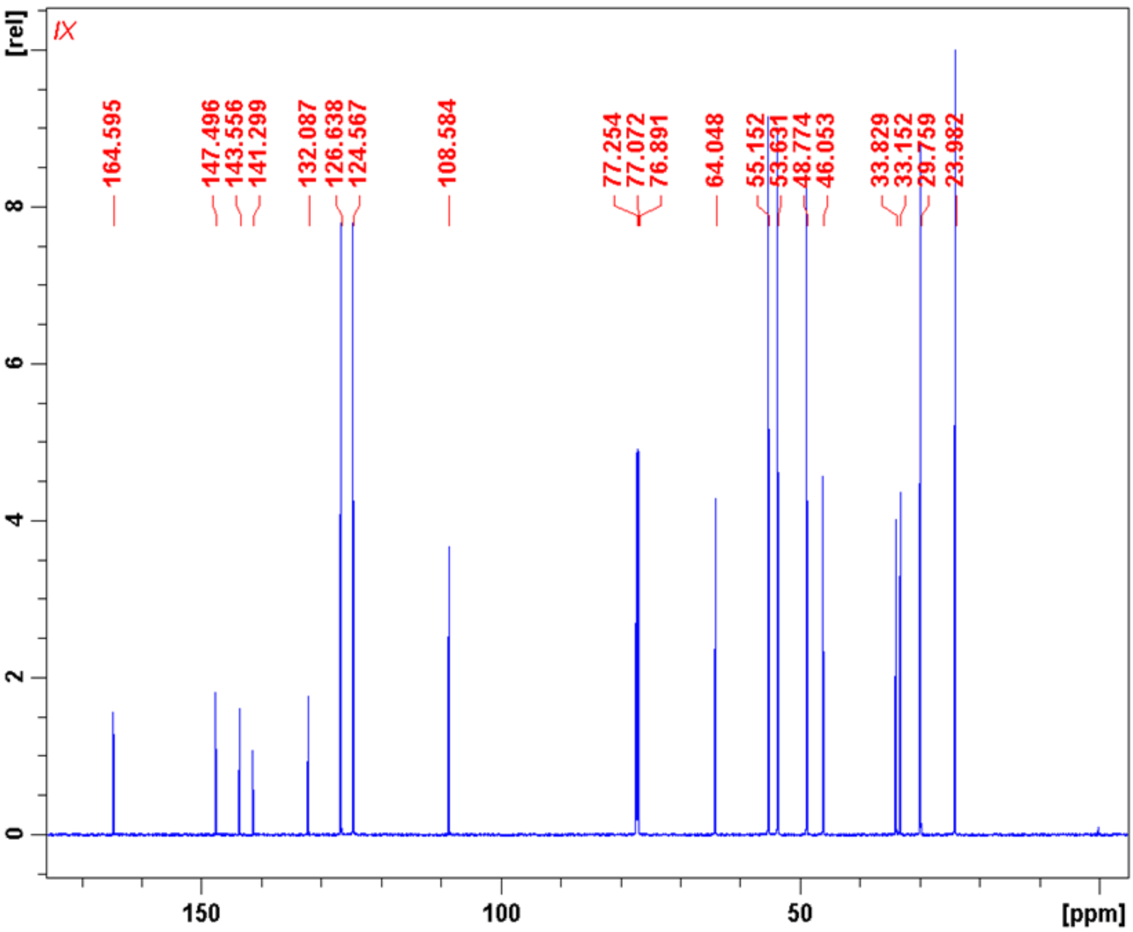

IX
164.595
147.496
143.556
141.299
132.087
126.638
124.567
108.584
77.254
77.072
76.891
64.048
55.152
53.631
48.774
46.053
33.829
33.152
29.759
23.982
[rel]
0
2
4
6
8
150
100
50
[ppm]

V.

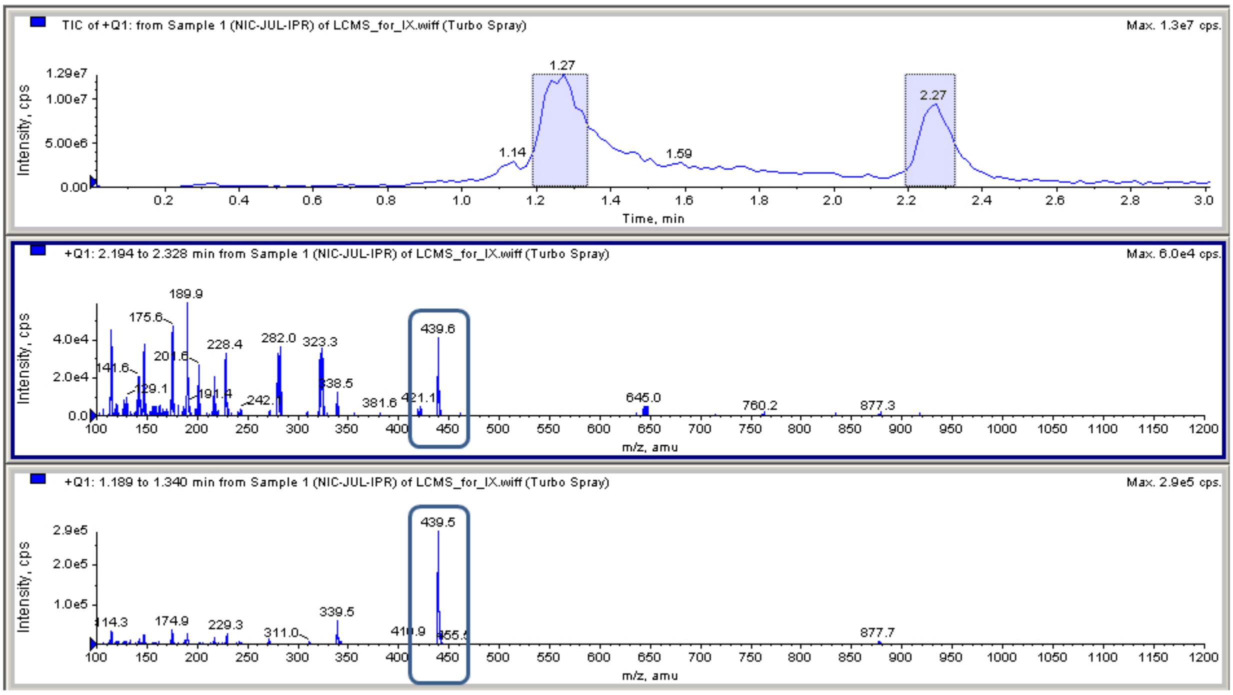

W.

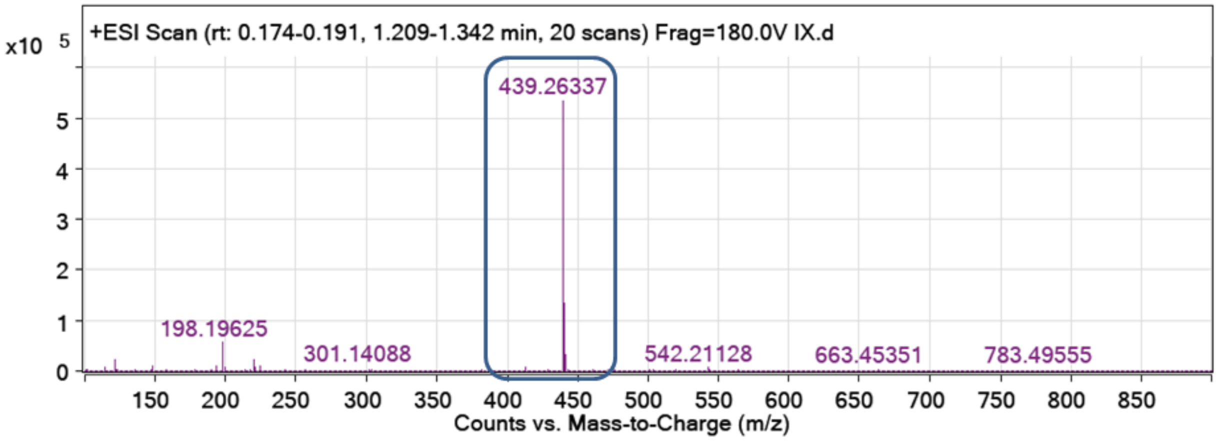

Supplementary figure 2. Chemical characterizations of E260 –tartrate and its synthesis intermediates.

(**A**) ^{1}H-NMR of compound II. **(B)** ^{13}C-NMR of compound II. (**C**) HRMS analysis of the compound II confirms a molecular weight of 334.20986 Dalton as calculated (Boxed with blue Square). (**D**) ^{1}H-NMR of compound III. **(E)** ^{13}C-NMR of compound

III. **(F)** HRMS analysis of the compound III confirms a molecular weight of 212.17620 Dalton as calculated (Boxed with blue Square) (**G**) ^{1}H-NMR of compound IV. (**H**) ^{13}C-NMR of compound IV. (**I**) HRMS analysis of the compound IV confirms a molecular weight of 198.19630 Dalton as calculated (Boxed with blue Square). (**J**) ^{1}H-NMR of compound Va. (**K**) ^{13}C-NMR of compound Va. (**L**) HRMS analysis of the compound Va confirms a molecular weight of 102.01207 Dalton as calculated (Boxed with blue Square). (**M**) ^{1}H-NMR of compound VI. (**N**) ^{13}C-NMR of compound VI. (**O**) HRMS analysis of the compound VI confirms a molecular weight of 181.92066 Dalton as calculated (Boxed with blue Square). (**P**) ^{1}H-NMR of compound VIII . Integral of peaks reflects number of protons (**Q**) ^{13}C-NMR of compound VIII. (**R**) LCMS analysis of compound VIII with a retention time of 2.08 and molecular weight of 322.2, 324.0 Dalton in 1:1 ratio (Boxed with blue square), due to^{79}Br, ^{81}Br isotopes.(**S**) HRMS analysis of the compound VIII confirms a molecular weight of 322.00047 Dalton as calculated (Boxed with blue Square). (**T**) ^{1}H-NMR of compound IX . Integral of peaks reflects number of protons.(**U**) ^{13}C-NMR of compound IX. (**V**) LCMS analysis of compound IX with two retention times of 1.27 as the main and 2.27 as an overload which integrated also into the second peak and a molecular weight of 439.5 Dalton (Boxed with blue square). (**W**) HRMS analysis of the compound IX confirms a molecular weight of 439.26337 Dalton as calculated (Boxed with blue Square).

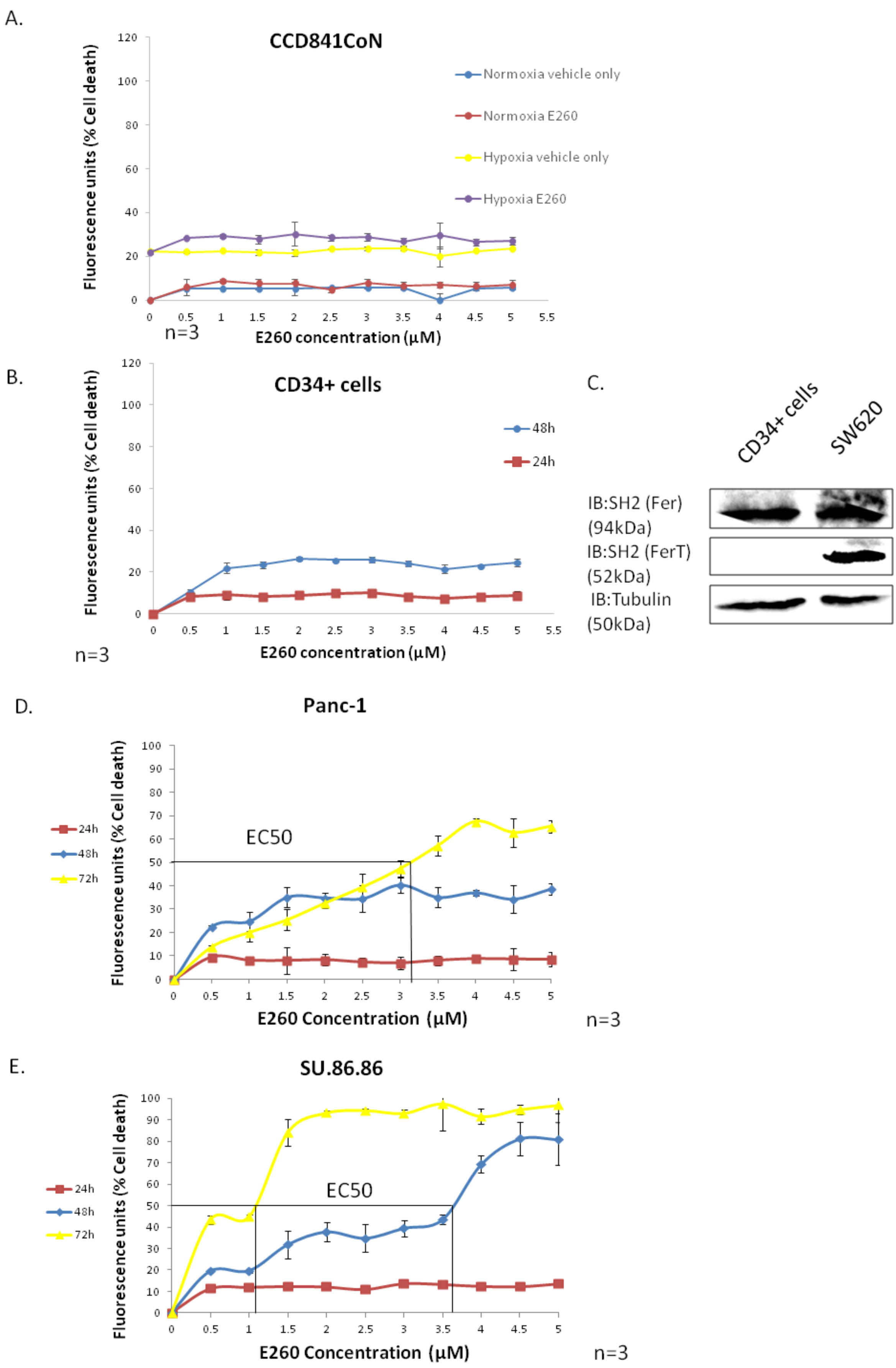
A.
CCD841CoN
Normoxia vehicle only
Normoxia E260
Hypoxia vehicle only
Hypoxia E260
Fluorescence units (% Cell death)
E260 concentration (μM)
n=3
B.
CD34+ cells
48h
24h
Fluorescence units (% Cell death)
E260 concentration (μM)
n=3
C.
CD34+ cells
SW620
IB:SH2 (Fer)
(94kDa)
IB:SH2 (FerT)
(52kDa)
IB:Tubulin
(50kDa)
D.
Panc-1
24h
48h
72h
EC50
Fluorescence units (% Cell death)
E260 Concentration (μM)
n=3
E.
SU.86.86
24h
48h
72h
EC50
Fluorescence units (% Cell death)
E260 Concentration (μM)
n=3

Supplementary figure 3. Effect of E260 on non-metastatic and metastatic pancreatic cancer cells, normal and hypoxic epithelial cells, and hematopoietic stem cells (CD34+). (**A**) Dose response curve depicting the death levels of normal CCD841CoN epithelial cells treated for 24h with either vehicle or ascending concentrations of E260 under normoxic or hypoxic conditions; n=3, +/- SE. (**A**) Dose response curve depicting the death levels of human donor derived CD34 positive hematopoietic stem cells (CD34+) treated with increasing concentrations of E260 for 24h (Red line) , or 48h (Blue line); n=3, +/- SE. (**C**) WB analysis of Fer and FerT protein levels in CD34+ and SW620 cells lysate.(**D**) Dose response curve depicting the death levels of non-metastatic pancreatic cancer cells- PANC-1, treated with increasing concentrations of E260 for 24h (Red line) ,48 h (Blue line) or 72h (Yellow line); n=3, +/- SE. (**E**) Dose response curve depicting the death levels of metastatic pancreatic cancer cells -SU.86.86, treated with increasing concentrations of E260 for 24h (Red line) ,48 h (Blue line) or 72h (Yellow line) ; n=3, +/- SE.

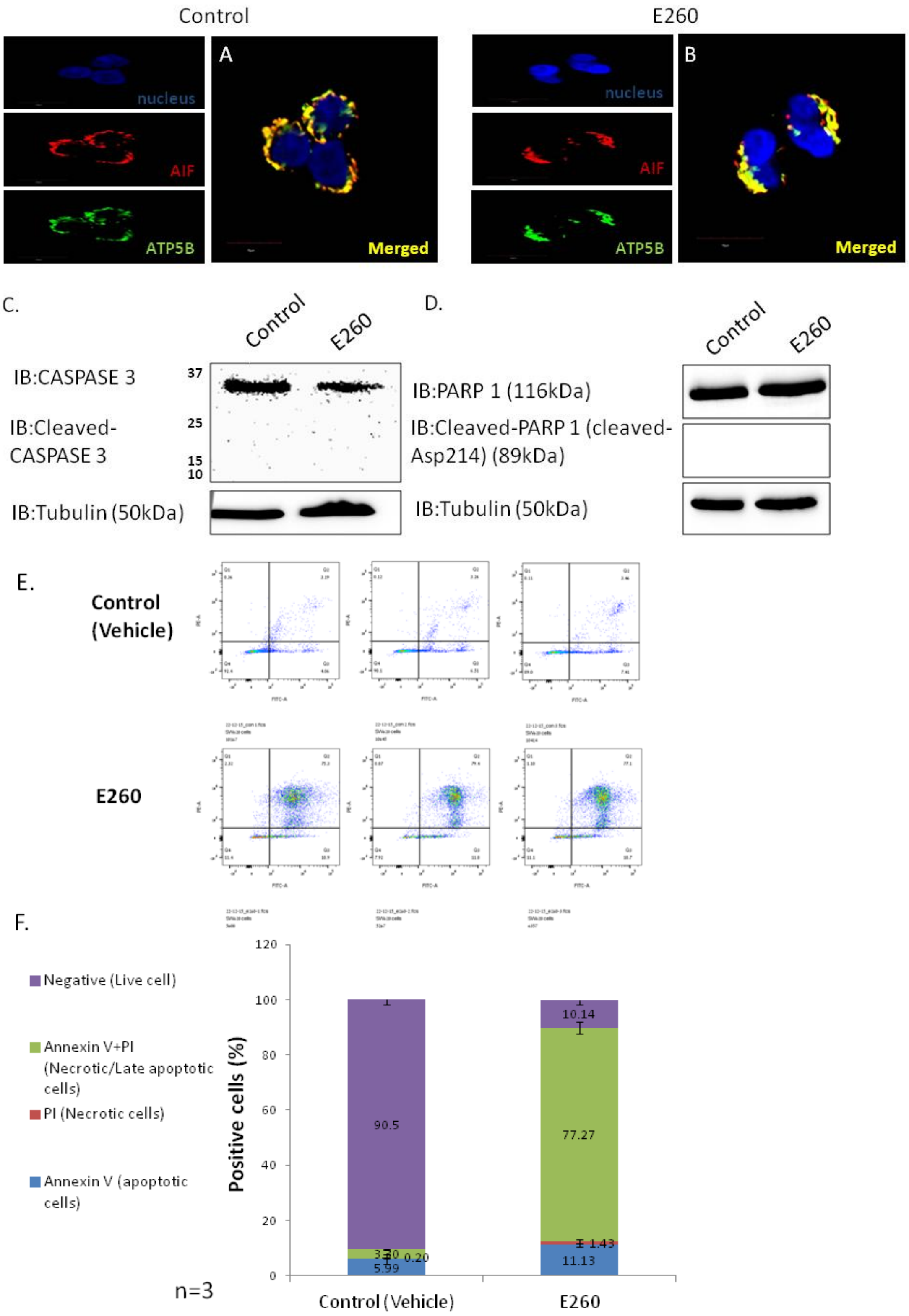

Supplementary figure 4. E260 evokes necroptosis and not apoptosis in treated CC cells. (**A and B**) Staining for AIF (Red) and ATP5B (Green) in SW620 cells treated with control vehicle (**A**) or E260 (**B**) for 16h. Separate channels are presented

on the left of each merged image. Yellow color represents co-localization. Bars represent 10 µm. Nuclei were visualized with Hoechst (Blue). (**C**) Protein lysates from SW620 cells treated with control vehicle or 2µM E260 for 16h, were subjected to WB analysis using anti-Caspase 3 and anti-tubulin antibodies. (**D**) Protein lysates from control or 2µM E260 treated SW620 cells were subjected to WB analysis using anti-PARP-1 and anti-tubulin antibodies. (**E-F**) FACS analysis of Annexin V-PI staining in cells treated with control solution (vehicle) or 2 µM E260 for 16h.

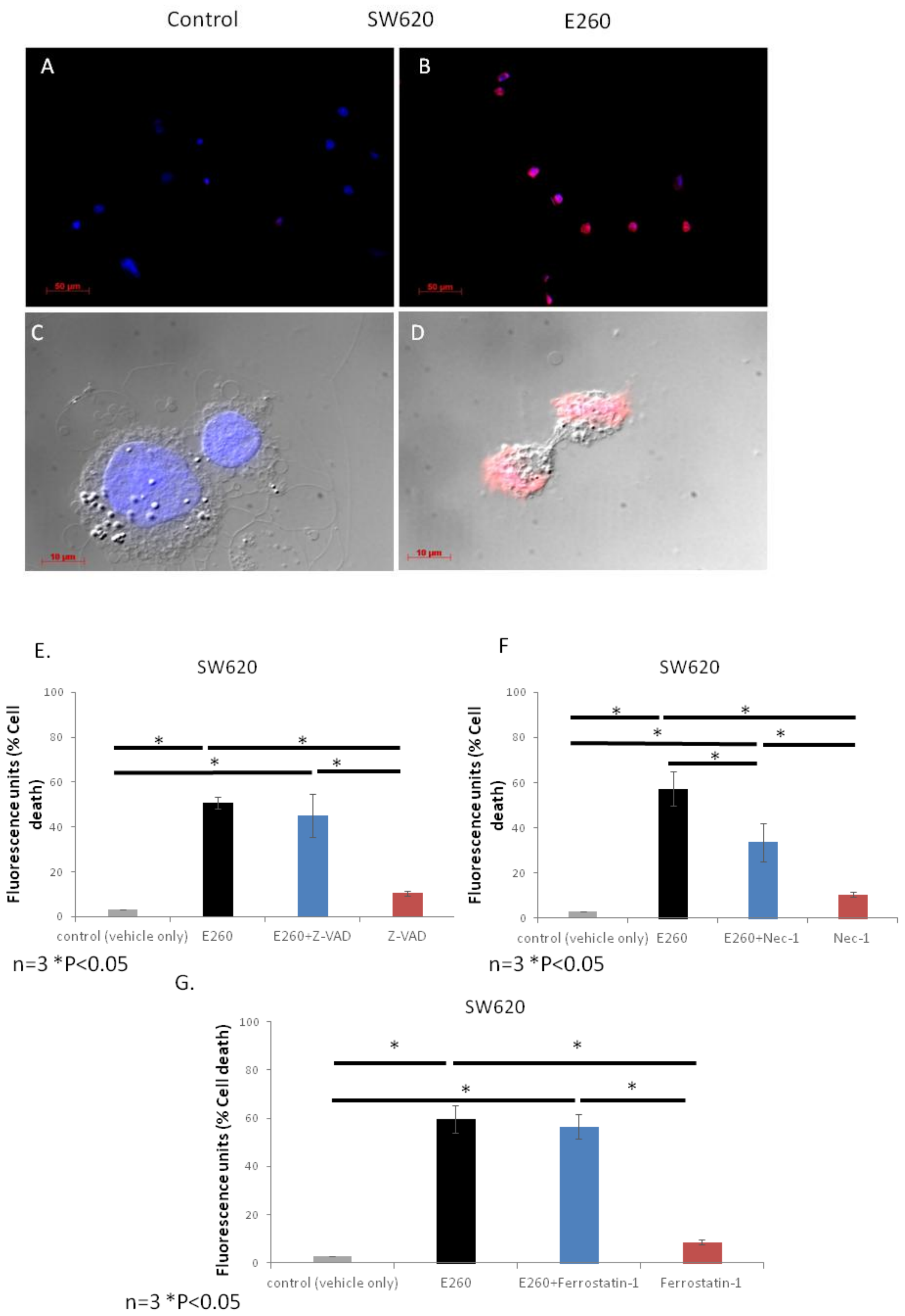

Supplementary figure 5. Apoptotis inhibitors does not affect E260 cytotoxicity in treated SW620 cells

(**A-D**) SW620 cells treated with vehicle (**A** and **C**), or E260 (**B** and **D**) for 16h, were subjected to EthIII dye (Red) and Hoechst (Blue) uptake assay and visualized under confocal fluorescence microscope to detect necrotic cells. Bars represent 50µm in **A** and **B**, and 10µm in **C** and **D**. (**E**) Cell death levels in SW620 cells treated for 16h with vehicle (Grey), 2µM E260 (Black), 2µM E260 and 50µM Z-VAD (Blue), or 50µM Z-VAD alone (Red); n=3, +/-SE. (**F**) Cell death levels in SW620 cells treated for 16h with vehicle (Grey), 2µM E260 (Black), 2µM E260 and 100µM Nec-1 (Blue), or 100µM Nec-1 alone (Red); n=3, +/-SE. (**G**) Cell death levels in SW620 cells treated for 16h with vehicle (Grey), 2µM E260 (Black), 2µM E260 and 1µM Ferroststin-1 (Blue), or 1µM Ferrostatin-1 alone (Red); n=3, +/-SE.

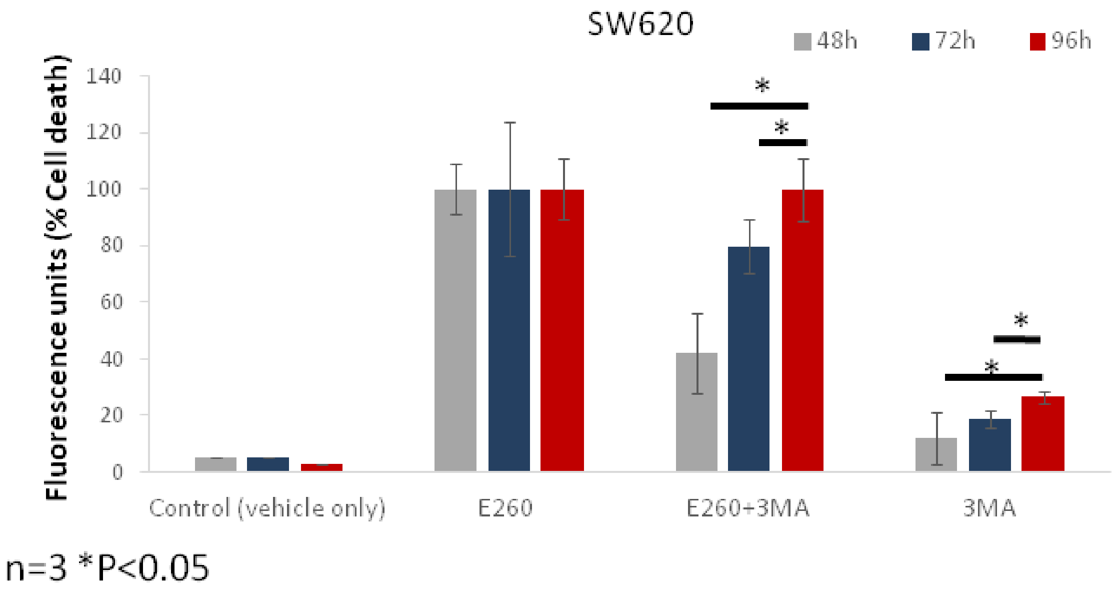

Supplementary figure 6. 3-MA attenuates the cytotoxic effects of E260. Cell death levels in SW620 cells treated for different periods of time with vehicle, 2µM E260, 2µM E260 and 5mM 3-MA, or 5mM 3-MA alone; n=3, +/-SE.

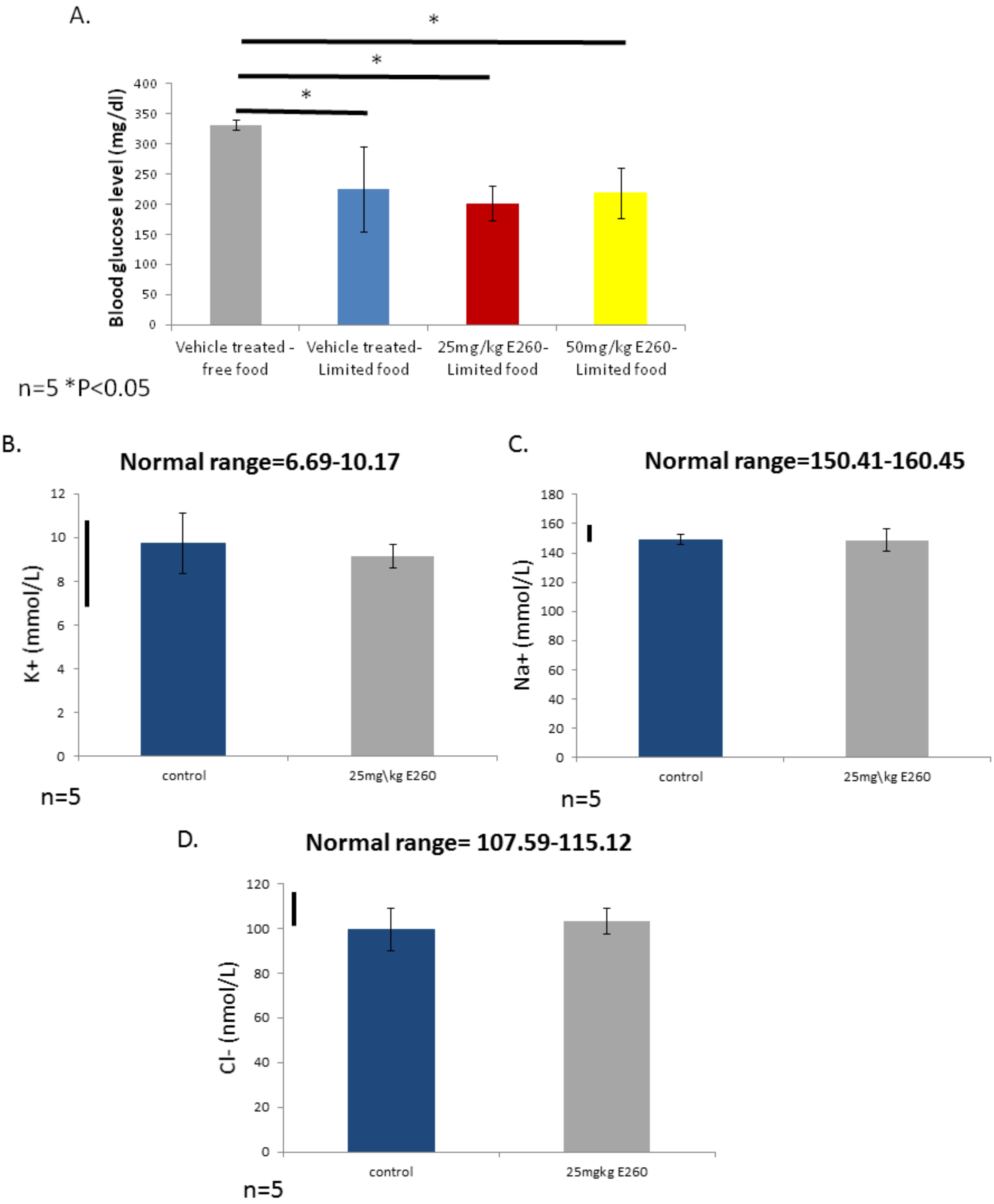

Supplementary figure 7. Blood Glucose and electrolyte levels of E260 treated mice. **(A)** Serum Glucose levels in mice treated with control vehicle and given *ad-libitum* food access (Grey), treated with control vehicle and given limited food access (Blue)**,** treated with 25mg/kg E260 and given limited food access (Red) and treated

with 50mg/kg E260 and given limited food access (Yellow). (**B-D**) Blood electrolyte levels in serum of control (Blue) and 25mg/kg E260 treated (Grey) mice; n=5, +/-SD.

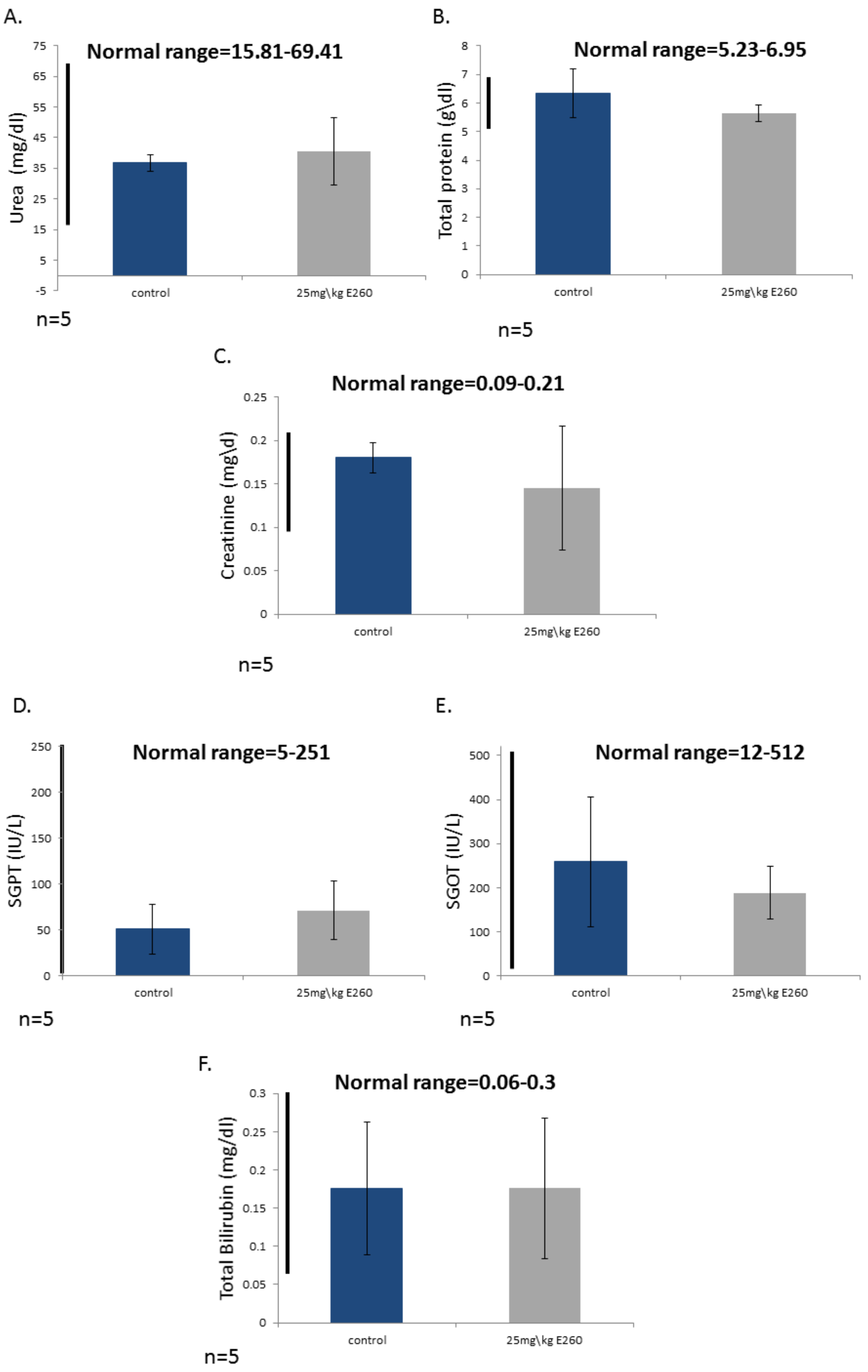

Supplementary figure 8. Blood Kidney and liver function markers levels of E260 treated mice. (**A-C**) Serum levels of kidney function markers in control (Blue) and

25mg/kg E260 treated (Grey) mice; n=5, +/-SD. (**D-G**) Serum levels of liver enzymes in serum of control (Blue) and 25mg/kg E260 (Grey) treated mice; n=5, +/-SD.

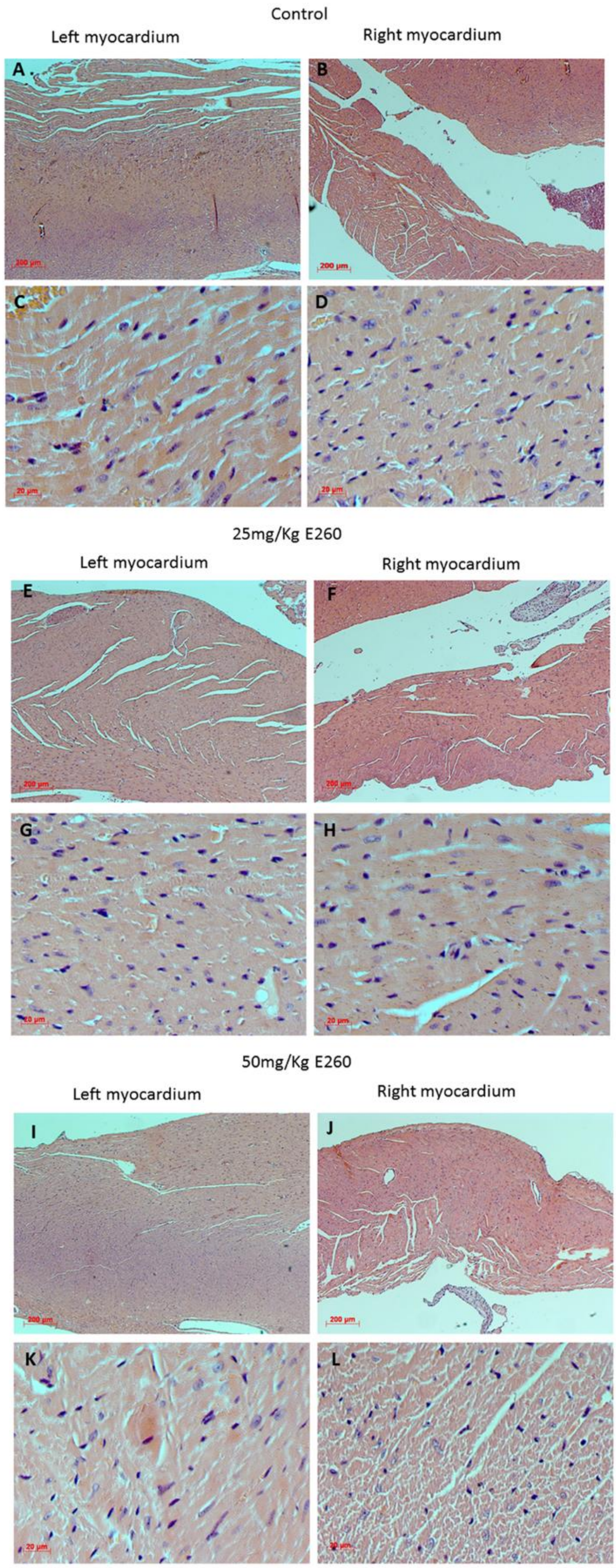
Control
Left myocardium
Right myocardium
A
B
C
D
25mg/Kg E260
Left myocardium
Right myocardium
E
F
G
H
50mg/Kg E260
Left myocardium
Right myocardium
I
J
K
L

Supplementary figure 9. Histopathological analysis of hearts from E260 treated mice. Hearts from control (**A-D**), 25mg/kg E260 (**E-H**), and 50mg/kg E260 (**I-L**) treated mice were fixed, embedded in paraffin, and stained with H&E for histopathological analysis. Five mice were examined from each group, and representative images are shown.

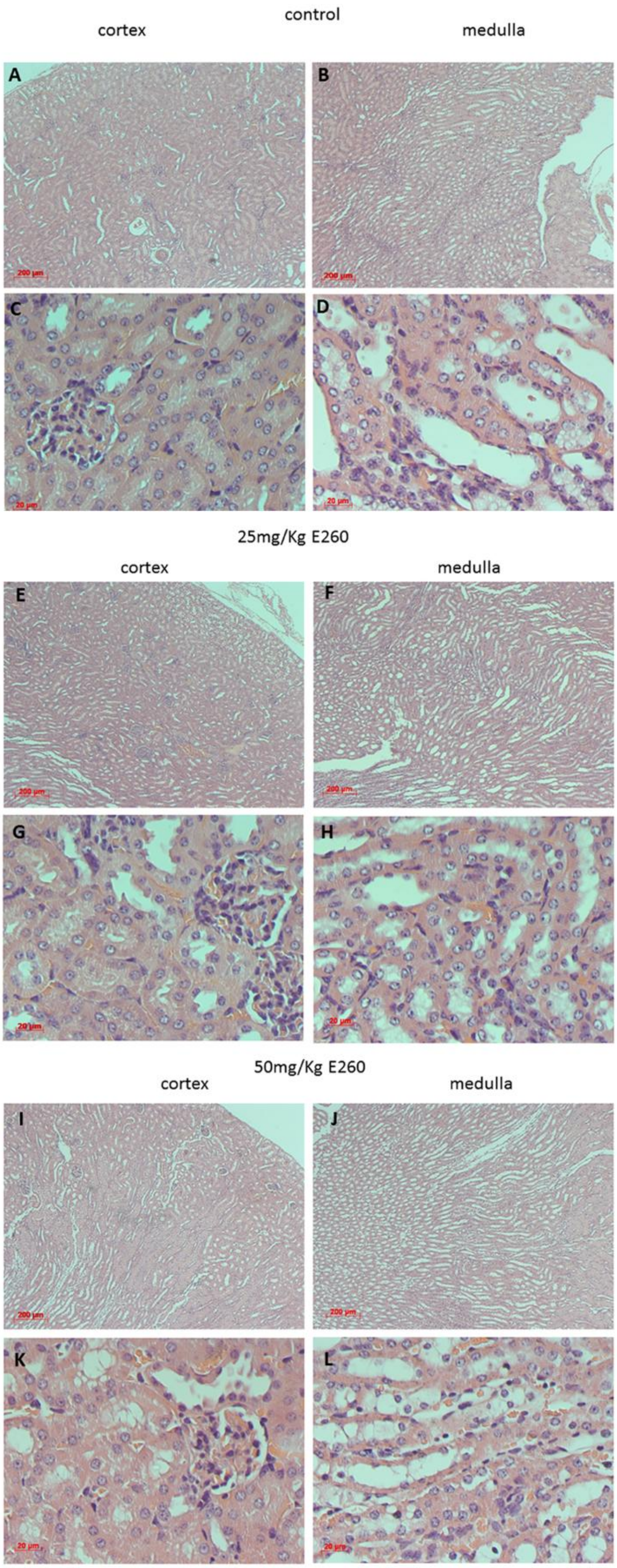
control
cortex
medulla
A
B
C
D
25mg/Kg E260
cortex
medulla
E
F
G
H
50mg/Kg E260
cortex
medulla
I
J
K
L

Supplementary figure 10. Histopathological analysis of kidneys from E260 treated mice. Kidneys from control (**A-D**), 25mg/kg E260 (**E-H**), and 50mg/kg E260 (**I-L**) treated mice were fixed, embedded in paraffin, and stained with H&E for histopathological analysis. Five mice were examined from each group, and representative images are shown.

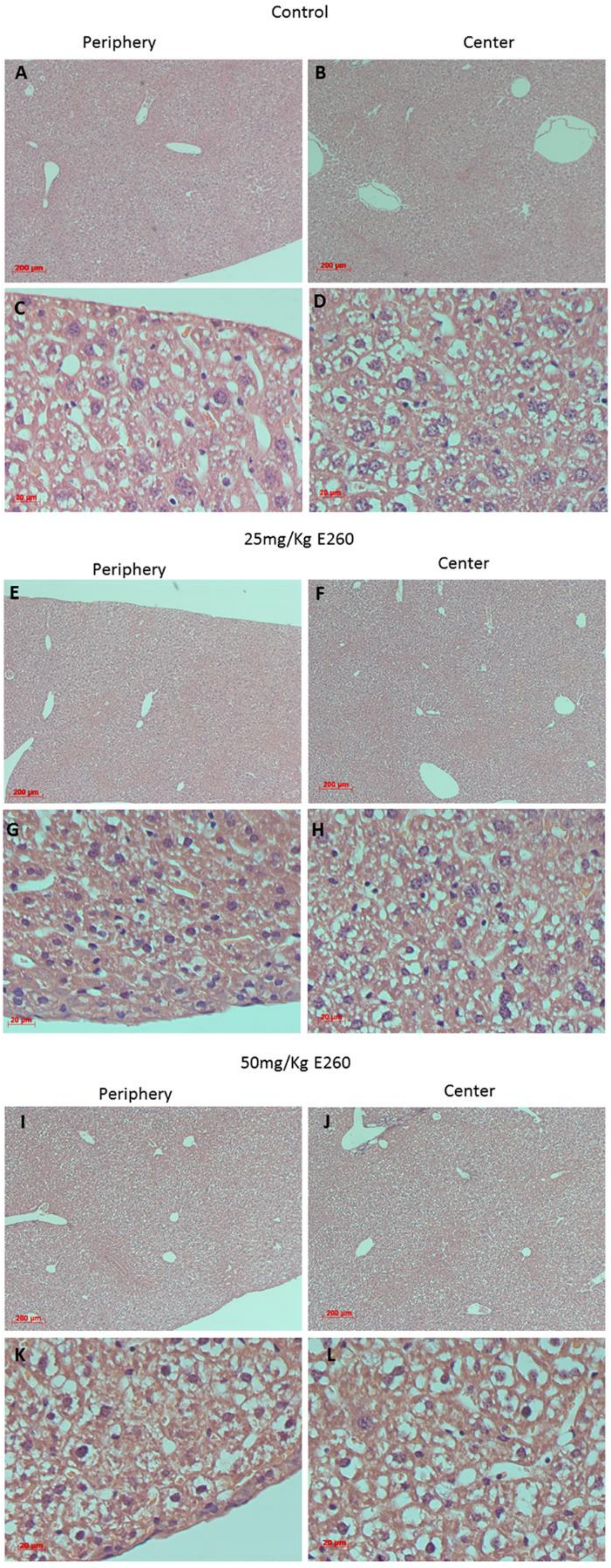
Control
Periphery
Center
A
B
C
D
25mg/Kg E260
Periphery
Center
E
F
G
H
50mg/Kg E260
Periphery
Center
I
J
K
L

Supplementary figure 11. Histopathological analysis of livers from E260 treated mice. Livers from control (**A-D**), 25mg/kg E260 (**E-H**), and 50mg/kg E260 (**I-L**) treated mice were fixed, embedded in paraffin, and stained with H&E for histopathological analysis. Five mice were examined from each group, and representative images are shown.

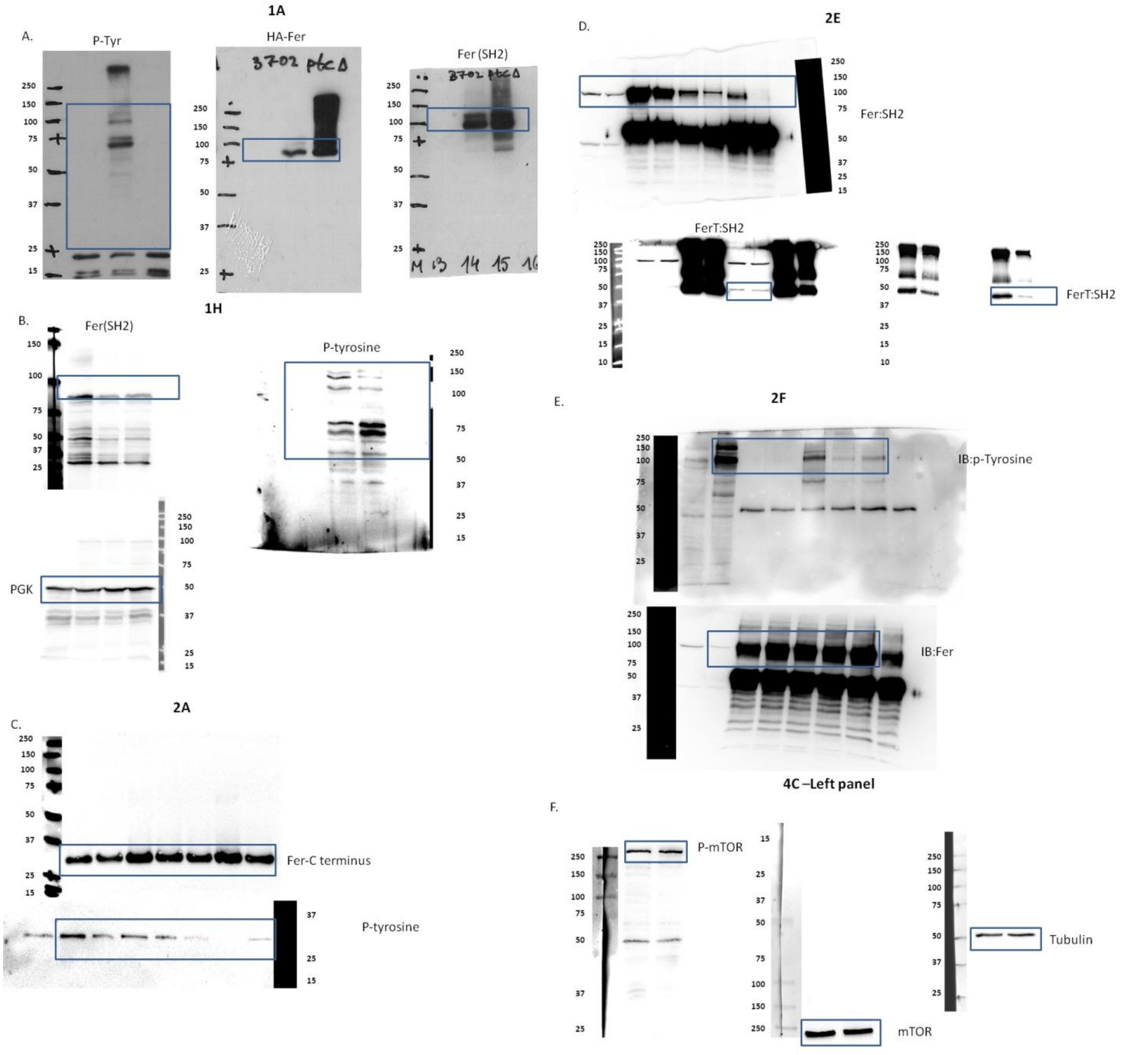

Supplementary figure 12.Uncropped full blots which appear in this manuscript. Cropped areas are boxed and labeled with the correlating protein. Figure number appears at the head of each blots panel. (A) Figure 1A. (B) Figure 1H. (C) Figure 2A. (D) Figure 2E. (E) Figure 2F. (F) Figure 4C- Left panel. Size markers (in kDa) are presented near each blot.

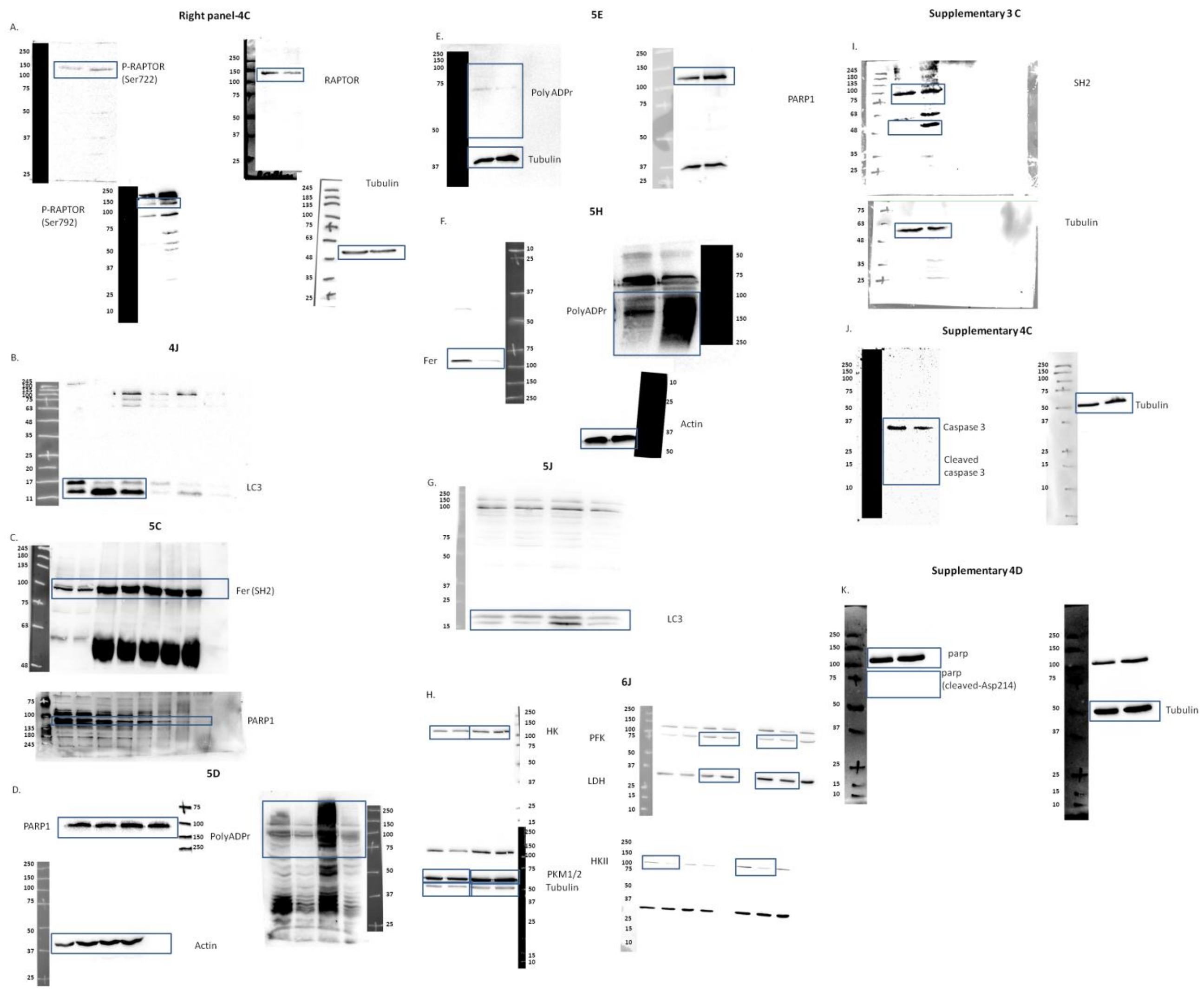

Supplementary figure 13.Uncropped full blots which appear in this manuscript. (A) Figure 4C- Right panel. (B) Figure 4J. (C) Figure 5C. (D) Figure 5D. (E) Figure 5E. (F) Figure 5H. (G) Figure 5J. (H) Figure 6J. (I) Supplementary figure 3C. (J) Supplementary figure 4C. (K) Supplementary figure 4D. Size markers (in kDa) are presented near each blot.
